# A Novel Molecular Solution for Ultraviolet Light Detection in Caenorhabditis elegans

**DOI:** 10.1371/journal.pbio.0060198

**Published:** 2008-08-05

**Authors:** Stacey L Edwards, Nicole K Charlie, Marie C Milfort, Brandon S Brown, Christen N Gravlin, Jamie E Knecht, Kenneth G Miller

**Affiliations:** Genetic Models of Disease Program, Oklahoma Medical Research Foundation, Oklahoma City, Oklahoma, United States of America; Cambridge University, United Kingdom

## Abstract

For many organisms the ability to transduce light into cellular signals is crucial for survival. Light stimulates DNA repair and metabolism changes in bacteria, avoidance responses in single-cell organisms, attraction responses in plants, and both visual and nonvisual perception in animals. Despite these widely differing responses, in all of nature there are only six known families of proteins that can transduce light. Although the roundworm Caenorhabditis elegans has none of the known light transduction systems, we show here that C. elegans strongly accelerates its locomotion in response to blue or shorter wavelengths of light, with maximal responsiveness to ultraviolet light. Our data suggest that C. elegans uses this light response to escape the lethal doses of sunlight that permeate its habitat. Short-wavelength light drives locomotion by bypassing two critical signals, cyclic adenosine monophosphate (cAMP) and diacylglycerol (DAG), that neurons use to shape and control behaviors. C. elegans mutants lacking these signals are paralyzed and unresponsive to harsh physical stimuli in ambient light, but short-wavelength light rapidly rescues their paralysis and restores normal levels of coordinated locomotion. This light response is mediated by LITE-1, a novel ultraviolet light receptor that acts in neurons and is a member of the invertebrate Gustatory receptor (*Gr*) family. Heterologous expression of the receptor in muscle cells is sufficient to confer light responsiveness on cells that are normally unresponsive to light. Our results reveal a novel molecular solution for ultraviolet light detection and an unusual sensory modality in C. elegans that is unlike any previously described light response in any organism.

## Author Summary

In all of nature, scientists have discovered only six different mechanisms by which organisms sense light, and only one of these mechanisms can detect ultraviolet light (the rhodopsins that sense ultraviolet light in non-mammalian vertebrates). The widely studied model organism Caenorhabditis elegans has none of the known light transduction systems, but we discovered that C. elegans has a robust locomotory response to ultraviolet light. C. elegans may use this light response to escape damaging or lethal doses of sunlight. Ultraviolet and other shortwave light, such as violet and blue wavelengths, drive locomotion by bypassing two critical signals, cyclic adenosine monophosphate (cAMP) and diacylglycerol (DAG), that neurons use to shape and control behaviors. C. elegans mutants lacking these signals are paralyzed and unresponsive to harsh physical stimuli in ambient light, but short-wavelength light rapidly rescues their paralysis and restores greater-than-normal levels of coordinated locomotion. This astonishing light response is mediated by a novel ultraviolet light receptor that acts in neurons. Our results reveal a novel molecular solution for ultraviolet light detection and an unusual sensory modality in C. elegans that is unlike any previously described light response in any organism.

## Abstract 3

An ultraviolet light receptor drives a robust locomotory response that restores normal levels of movement to paralyzed neuronal signaling mutants. This may help*C. elegans* avoid lethal doses of sunlight.

## Introduction

An animal's complement of sensory abilities reflects its unique evolutionary and natural history. Despite its status as a major model organism, little is known about the natural history of Caenorhabditis elegans. Past studies have revealed four major modalities through which C. elegans senses its environment: chemosensation, mechanosensation, osmosensation, and thermosensation [1–4]. These four modalities would seem sufficient to meet the sensory needs of what is often referred to as a subterranean animal; however, recent studies suggest that C. elegans may spend much of its time above ground, living on small surface-dwelling animals or their carcasses [5,6]. C. elegans may therefore be frequently exposed to direct sunlight, which can damage or kill cells by photo-oxidative reactions [7]. If C. elegans spends significant time above ground, it would need a sensory mechanism for detecting and avoiding lethal doses of direct sunlight. However, despite extensive observation under blue, blue-violet, and even ultraviolet (UV) light, there are no published reports of a behavioral response to high energy light (blue wavelengths or shorter) in C. elegans. Here we show that C. elegans does in fact have a strong response to short wavelength light that takes the form of a robust acceleration of locomotion. Our data suggest that C. elegans uses this light response to escape the ultraviolet light in direct sunlight. This light response can restore normal or hyperactive locomotion to certain kinds of paralyzed synaptic signaling mutants. To identify the molecular basis for this form of light reception, we performed a forward genetic screen and identified mutants defective in the response. The mutations disrupted LITE-1, which is a member of the Gustatory receptor (*Gr*) family that we show functions as a short wavelength light detector when expressed in a heterologous tissue.

## Results

### A Light Response Restores Movement to Paralyzed Gα Signaling Mutants

**Figure 1:**
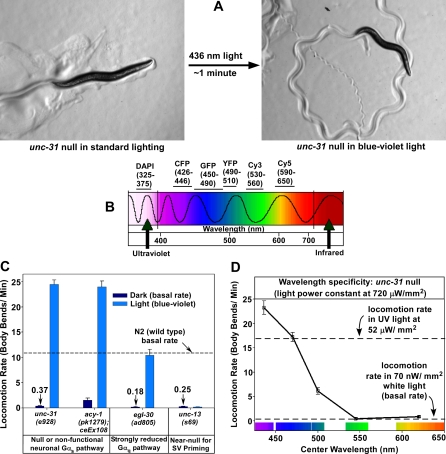
Blue Light–Mediated Rescue of Paralyzed Synaptic Signaling Mutants (A) Images show an *unc-31* null mutant on a bacterial lawn before and after illumination with 436-nm light. (B) Filters for determining the wavelength specificity of the light response. Lines above the visible light spectrum indicate the wavelength range of light that each filter transmits to the microscope objective (numbers are wavelengths in nanometers). The filter names refer to chromophores that are irrelevant to these studies. Chroma filter numbers (from lowest to highest wavelengths): 31000v2, 31044v2, 41017, 41028, 41007a, and 41008. (C) Mean locomotion rates of representative synaptic signaling mutants in low white light versus blue-violet light of optimal power (1460 μW/mm^2^). Numbers above some bars state the precise locomotion rates. Error bars are the standard errors of ten animals. (D) Mean locomotion rates of an *unc-31* null mutant illuminated with various wavelengths. Error bars are the standard errors of ten animals.

Cyclic AMP (cAMP) and diacylglycerol (DAG) are important universal signals that neurons use to shape and drive behaviors, learning, and memory. Neurons use Gα proteins to tightly control the production of cAMP and DAG at synapses. In C. elegans, convergent Gα_q_ and Gα_s_ pathways make DAG and cAMP, respectively, which control synaptic activity to generate the locomotion behavior (Figure S1). Mutations that eliminate either of these two major pathways result in animals that are nearly paralyzed and are essentially unresponsive to harsh physical stimulation (Videos S1 and S3). However, blue-violet light projected onto paralyzed *unc-31* null mutants, which have a nonfunctional Gα_s_ pathway [8], restored coordinated locomotion (Figure 1A and Video S2) and increased their mean locomotion rate over a 6-min period to a level that was 65-fold higher than the basal rate of the mutant and 2-fold higher than the basal rate of wild type (Figure 1C). Blue-violet light caused a similar increase in the locomotion rate of an *acy-1* (adenylyl cyclase) null mutant that lacks all Gα_s_-driven locomotion specifically in neurons [9,10] (Figure 1C). Thus, the light response pathway does not require the production of cAMP through the neuronal Gα_s_–adenylyl cyclase pathway. Blue-violet light also restored coordinated locomotion to a strong reduction-of-function *egl-30* (Gα_q_) mutant (Video S4) and increased its locomotion rate 67-fold, to a level that was not significantly different from the basal rate of wild-type animals. Thus, the light response pathway, at the most, requires only very low levels of the DAG and activated RhoA produced by the Gα_q_ pathway. In contrast, blue-violet light did not affect the movement of a similarly paralyzed *unc-13* mutant, which has defects in the late stages of neurotransmitter release (Figure 1C). Thus, the light response requires neurotransmitter release and does not induce nervous system–independent muscle activity.

### Wavelength Specificity of the Response

We determined the wavelength specificity of the response by projecting light through a series of filters in a stereomicroscope nosepiece (Figure 1B) and measuring the locomotion response of the *unc-31* mutant at various wavelengths and a constant light power of 720 μW/mm^2^. At this power, the mutant only responded to wavelengths of ∼500 nm (blue-green) or shorter (Figure 1D). The fold-increase over the basal response increased from zero at 545 nm (green) to ∼65-fold at 441 nm (blue-violet). Although the maximum ultraviolet (UV) power produced by this light system was 52 μW/mm^2^, UV light of this power caused a response equal to 720 μW/mm^2^ of blue light (Figure 1D). Thus, the light response is most sensitive to UV light, but higher levels of violet and blue light also activate it. Wild-type animals had the same wavelength sensitivity as the *unc-31* mutant, although wild type's peak locomotion rate in 720-μW/mm^2^ blue violet light was ∼1.5-fold higher than the *unc-31* null mutant (Figure S2).

### Relationship of Light Dose to Response at Various Wavelengths

**Figure 2:**
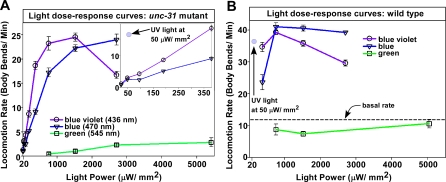
The Relationship of Light Dose to Locomotion Rate at Various Wavelengths (A) Mean locomotion rate of the *unc-31(e928)* mutant at various powers and wavelengths of light. The highest power for each color is close to the maximum power of that color that we can project onto the culture plate. The maximum power of green light causes only a weak response. The inset expands the low power region of the graph and shows the strong response to UV light at a power where blue and blue-violet light cause only weak responses. Error bars in (A) and (B) are the standard errors in populations of ten animals. (B) Response of N2 (wild type) to various powers and wavelengths of light. A dashed line indicates N2′s basal rate in low power white light (70 nW/mm^2^). Note that low power UV light causes a response greater than a 7-fold higher power of blue or blue-violet light. Note that green light does not cause a significant locomotion response, even at the highest power.

A comparison of the light responses at various powers of blue-violet, blue, and green light again highlighted the strong specificity of the response for short wavelengths, especially ultraviolet light. Increasing the power of green light to 5,500 μW/mm^2^ only improved the locomotion rate of the *unc-31* null by 6.7-fold (Figure 2A) and did not affect the locomotion rate of wild type (Figure 2B). In contrast, the blue and blue-violet responses of the *unc-31* mutant increased sharply between 50 and 700 μW/mm^2^, peaking at 1,400 and 2,800 μW/mm^2^ for blue-violet and blue light, respectively. At these powers, both blue and blue-violet light increased the locomotion rates of wild-type and *unc-31* mutants 3.5- and 67-fold, respectively. At blue-violet powers greater than 1,400 μW/mm^2^ the locomotion rate decreased significantly from its peak level (Figure 2A), possibly due to overstimulation of the response or light damage over the 6-min assay period (more on this below). Wild-type animals required about half as much light power as the *unc-31* null mutants to maximize their responses to blue and blue-violet light (Figure 2B). The dose-response data also showed that a UV power of 50 μW/mm^2^ produces a response that is about equal to 350 μW/mm^2^ blue-violet light. Thus, UV light is about 7-fold more potent than blue-violet light in producing the response. The white light power of ambient room lighting is about 0.5 μW/mm^2^, which is 100-fold weaker than the minimum power necessary to induce the light response (Table S1). Thus, this light response is optimized for high powers of ultraviolet light.

### The Response to Light Does Not Correlate with Temperature Changes Induced by the Light

**Figure 3:**
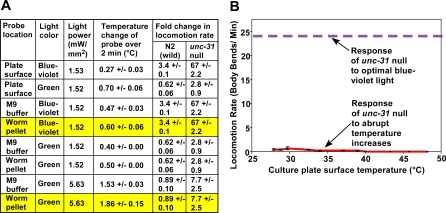
The Response to Light Is Not a Response to Temperature Changes Induced by the Light (A) The magnitude of the locomotory response to light does not correlate with light-induced temperature changes. We focused a light beam of the indicated color and power onto a metal temperature probe and noted the temperature change after 2 min of illumination. “Plate surface” means that the probe rested on the surface of the agar culture plate as we directly illuminated it. “Worm pellet” means the probe was inserted into a worm pellet of defined size and shape, consisting of living, assay-stage animals (see Materials and Methods). The worm pellet blocked direct light from reaching the probe, such that all heat effects are due to the light adsorption characteristics of the worms. Yellow highlighting indicates the essential data, which are the mean +/− standard error of three trials. All locomotion data for “Fold change in locomotion rate” are from Figure 2, except the data for N2 in green light. Note that “Fold change…” values of <1 indicate that the locomotion rate is lower than the locomotion rate in low white light. Errors for “Fold change in locomotion rate” are the standard errors of ten animals each. (B) Abruptly heating *unc-31* nulls does not make them move. We transferred *unc-31* nulls from a room temperature culture plate to a preheated culture plate and measured their locomotion rate in the first minute after transfer. Temperatures are an average of the plate surface temperatures at 30 and 90 s after transfer, which is the time period of the assay. Error bars are the standard errors of ten animals. Data for the response to optimal blue-violet light (dashed line) are from Figure 2.

The high powers of light required to elicit this response raise the possibility that the animals are responding to temperature changes induced by the light. However, when we inserted a temperature probe into a pellet of adult worms, such that the probe could only be heated by the interaction of light with the worms, the temperature changes induced by blue-violet and green light were not statistically different, and amounted to less than 0.7 °C (Figure 3A). Moreover, increasing the power of green light to a level that raised the temperature of the worms by 1.9 °C had no effect on their movement when we reproduced this power in a locomotion rate assay. In contrast, a much lower power of blue-violet light increased wild type's movement almost 3.5-fold (Figure 3A). Thus, there is no correlation between the small temperature changes induced by the light and the behavioral response. Furthermore, when we transferred paralyzed *unc-31* mutants from room temperature plates to culture plates preheated to various temperatures between 24 °C and 50 °C, and assayed them for locomotion rate in the first minute after transfer, we found no temperature that increased their locomotion rate (Figure 3B). Thus, abrupt temperature increases do not make paralyzed *unc-31* mutants move.

### Time Course of Light-Induced Movement

**Figure 4:**
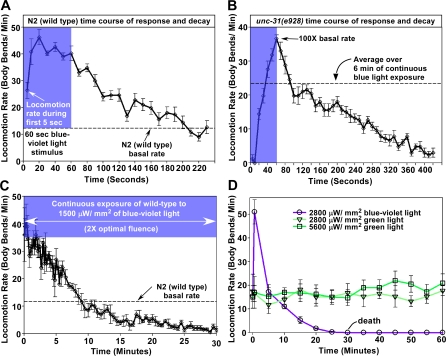
Time Course of Light-Induced Movement and Death (A) and (B) Mean locomotion rates of wild type and the *unc-31* null mutant during a time course exposure to blue-violet light of optimal power (730 μW/mm^2^ for wild type and 1460 μW/mm^2^ for the *unc-31* null). Blue-violet region indicates the time of illumination; later times show the response's decay after switching to low power white light. Error bars are the standard errors of ten animals. (C) and (D) After mounting a robust escape response, wild-type animals gradually slow down and die during continuous blue-violet illumination. Note that green light of twice this power does not cause death or even slow locomotion, even though it heats worms three times as much as the power of blue-violet light used in (C) (see Figure 3 for temperature measurements). Error bars are the standard errors of four animals.

To determine the time course of the light response, we measured locomotion rates at 5-s intervals during the first 10 s of light exposure, and then at 10-s intervals thereafter, and we blocked illumination at 1 min to observe the time course of decay. In blue-violet light, wild-type worms showed a rapid response initiation (1–2 s) and a slow response decay after turning off the light (∼2 min). Their average locomotion rate in the first 5 s of exposure was 2-fold higher than the basal rate (Figure 4A). Their response peaked at 20 s, and, after turning off the light at 1 min, their locomotion slowly decayed to the basal rate over the next 140 s (Figure 4A). In contrast to wild type, the *unc-31* null showed no response during the first two 5-s intervals but rapidly accelerated thereafter, until after 1 min, its locomotion rate was 100-fold higher than its basal rate (Figure 4B). After turning off the light, the *unc-31* null mutant took about twice as long as wild type for its response to decay to basal levels (Figure 4B). The delayed response of the *unc-31* null mutant, as well as the long decay times of both wild type and the mutant, suggests that light induces the build-up of a signal, and that the signal must reach higher levels in the mutant to induce the response.

### Time Course of Light-Induced Death

The *unc-31* mutant's peak locomotion rate after only 1 min of illumination was about 50% higher than its average rate over 6 min of continuous light exposure of the same power. This suggests that worms slow down over longer periods of illumination, and extended time course assays showed that to be true. Wild-type worms exposed to 1,500 μW/mm^2^ blue-violet light (i.e., twice the power that maximizes the locomotion response) over a 30-min illumination period steadily slowed down after their responses peaked (Figure 4C). This is because the animal is acutely injured by light exposure; it is not an adaptive response, because extended illumination with 2,800-μW/mm^2^ blue-violet light caused death in 25 min (Figure 4D). However, green light of the same power, or even double this power, did not kill worms and did not even affect their locomotion rate over a 1-h illumination period (Figure 4D). This suggests that these high powers of green light do not damage worms and that there is a sharp energy cutoff between blue and green wavelengths for heat-independent biological light damage. The light-induced death is not caused by overactivation of the light response, because mutants lacking the response (described below) die at the same time as wild-type animals during blue-violet light exposure (Figure S3).

### The C. elegans Light Response May Be a Photophobic Response to the UV Light in Direct Sunlight

**Figure 5:**
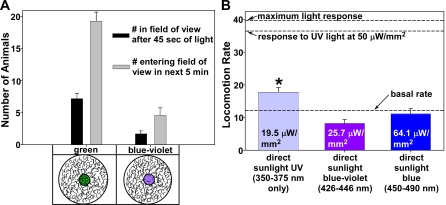
The C. elegans Light Response may be a Photophobic Response to the UV Light in Direct Sunlight (A) Worms actively avoid blue-violet light. Field-of-view clearing assays on crowded plates of N2 (wild type) in green versus blue-violet light. Each plate contained 4,500 young adult animals at a density of 1.6 animals/mm^2^. Error bars are the standard errors from ten trials. (B) The UV light in direct sunlight is sufficient to cause a locomotory response. Shown are the mean locomotion rates of wild type in ultraviolet, blue-violet, and blue light of powers found in direct sunlight. Note that the UV power measured by the light detector at the culture plate surface is restricted to wavelengths in the 350–375-nm range due to adsorption of lower wavelengths by glass in the microscope objective. Therefore, the power reading of 19.5 μW/mm^2^, taken at the culture plate surface, was biased toward longer wavelengths of the DAPI filter (350–375 nm). Sunlight passing through the DAPI filter directly onto the detector contains all wavelengths from 325–375 nm (because it does not go through glass), so the sunlight power reading of 19.5 μW/mm^2^ reflects both short and long wavelengths of UV. It is likely that the shorter wavelengths of UV light in sunlight, including wavelengths shorter that those passed by the DAPI filter, are required to maximize the locomotion response. Indeed, sunlight UV wavelengths down to 291 nm reach Earth's surface [7], and this is well below the range of the DAPI filter. The asterisk indicates statistical significance, with a *p-*value of 0.015 using the unpaired *t*-test with the Welch correction. Error bars are the standard errors in populations of ten animals.

Short wavelength–light kills worms, which suggests that the locomotion response is an escape strategy. To test this, we illuminated crowded plates containing defined numbers of animals and quantified the number of animals that remained in the illuminated area after 45 s of light, and the number of animals that fully entered the illuminated area over the next 5 min. Our results clearly showed that the response to light is a photophobic response, because animals actively avoided the blue-violet illuminated area (Figure 5A).

To test the hypothesis that avoidance of direct sunlight is the ecological reason that C. elegans has a light response, we used blue, blue-violet, and UV excitation filters to measure the power of these colors in direct sunlight at solar noon at an altitude of 1,276 feet (389 m) above sea level. Figure 5B shows the sunlight values for each color after correcting for the percent transmission of each filter. We reproduced these values on the stereomicroscope, and then tested the response of wild-type animals to these different wavelengths at these powers. This experiment could only reproduce the long-wavelength UV light in sunlight, because the glass in the microscope objective blocks all UV light below 350 nm. Since our wavelength-sensitivity curves showed potency increasing dramatically as the wavelength shortens, it is likely that the experiment underestimates the animal's true response to sunlight, since it cannot measure the response to UV wavelengths shorter than 350 nm, and sunlight at Earth's surface contains UV wavelengths down to 291 nm [7]. Despite this limitation, when we reproduced the power for direct sunlight long wavelength UV light (>350 nm), we observed a significant locomotion response, whereas the sunlight powers of blue and blue-violet light produced no response (Figure 5B). We therefore hypothesize that the C. elegans light response has evolved as a photophobic response to the ultraviolet light in direct sunlight, but that higher powers of blue-violet and blue light can also evoke the response.

### Identifying the Ultraviolet Light–Sensing Protein

To identify the ultraviolet light receptor, we performed a forward genetic screen to look for mutants that are not paralyzed, but are unresponsive to short wavelength light. We tested ∼250,000 grandprogeny of ethyl methane sulfonate (EMS)–mutagenized animals for their light responses, which represents 24-fold knockout coverage for an average protein [11] and found 20 light-unresponsive (Lite) mutants. These mutants represent one major gene target (18 alleles), which we named *lite-1*, and two very rare targets (1 allele each), which we named *lite-2* and *lite-3*. The current study focuses on *lite-1*.

**Figure 6:**
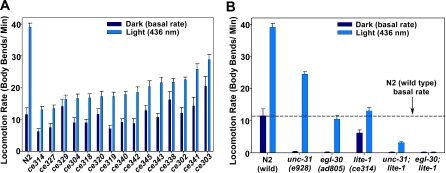
LITE-1 Mutations Strongly Reduce the Light Response and the Light-Driven Rescue of Synaptic Signaling Mutants (A) The strongest *lite-1* mutants, which are likely nulls, still have a residual response to light. Shown are the mean locomotion rates of wild type and the 15 unique *lite-1* mutants in low power white light (dark bars) or blue-violet light that maximizes the wild-type response (730 μW/ mm^2^ ;light bars). Error bars are the standard errors in populations of ten animals. (B) LITE-1 is required for light-driven rescue of synaptic signaling mutants. Shown are the mean locomotion rates of each strain during a 6-min exposure to low power white light (dark bars) or to an optimal power of blue-violet light (730 μW/mm^2^ for N2 and *lite-1(ce314)* and 1460 μW/mm^2^ for *unc-31* and *egl-30* single and double mutants). Error bars are the standard errors in populations of ten animals.

*lite-1* null mutants illuminated with optimal blue-violet light often showed no response to the light, but they responded normally to physical stimulation with a platinum wire (Videos S5 and S6). However, although some of the mutations should completely eliminate LITE-1′s function, even the strongest *lite-1* mutants still showed a residual response to light (Figure 6A). Although these data show that worms have a LITE-1–independent mechanism for responding to light, LITE-1 is clearly part of the major light response pathway. LITE-1 also has a major role in the light responses of the paralyzed synaptic signaling mutants. Without LITE-1, the *unc-31* mutant responds only weakly to light, and the Gα_q_ mutant shows no detectable response (Figure 6B).

**Figure 7:**
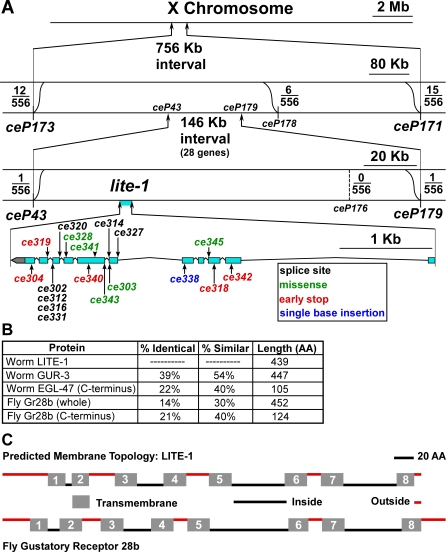
*lite-1* Mutations Disrupt an Eight-Transmembrane Protein with Homology to Insect Gustatory Receptors. (A) Mapping data. Scaled low to high resolution drawings show SNP markers used to map the mutations. Supplementary Table 2 precisely describes each mutation. (B) LITE-1 is similar to two other C. elegans proteins and fly gustatory receptors. (C) LITE-1 has a predicted membrane topology that is similar to its closest fly paralog. Scale drawings show TMHMM2.0–predicted transmembrane regions [62].

To find the molecular basis of the light response, we mapped the *lite-1* mutations to a 146-Kb interval containing 28 genes on the X chromosome (Figure 7A) and identified *lite-1* using candidate gene sequencing. Among the 18 *lite-1* alleles, our genetic screen produced seven splice site mutations, five early stop codons, five amino acid substitutions, and a single base insertion (Figure 7A and Table S2). LITE-1 (NCBI Protein Database (http://www.ncbi.nlm.nih.gov/sites/entrez?db=protein) accession number NP_509043.3) is a 439–amino acid protein related to the insect Gustatory receptor (*Gr*) family. Two other homologs complete the *C. elegans Gr* family: GUR-3 (NP_509743.2; 39% identical) and EGL-47 (NP_001023728.1; 22% identical in the C-terminal 105 residues). In flies, the *Gr* family has at least 68 members [12–14]. Sequence conservation within this family is low (15%–25%); however, a region near the C terminus including the last transmembrane domain is more highly conserved [15–17]. When compared to this family, LITE-1 shows the highest homology to *Drosophila Gr28b* (NP_995640.1), for which no function has been reported. Although LITE-1 and *Gr28b* are paralogs rather than orthologs, they are of similar lengths and have similar spacing of their transmembrane domains (Figure 7B and 7C). They are most homologous near their C termini (26% identical and 47% similar over a 68–amino acid stretch; Figure S4). Interestingly, transmembrane topology algorithms predict 8-transmembrane domains with extracellular N and C termini for both proteins (Figure 7C). Four of the five LITE-1 missense mutations disrupt residues within the transmembrane domains (Figure S4).

### LITE-1 Can Drive Light-Induced Locomotion When Expressed in all Neurons or Just in a Subset of Motor Neurons

**Figure 8:**
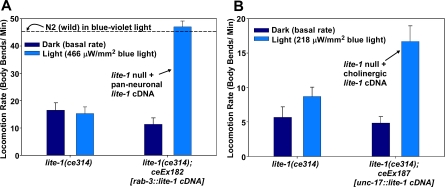
Expressing LITE-1 Pan-Neuronally Fully Rescues the Light Response, Whereas Expression Only in Cholinergic Motor Neurons Confers Partial Rescue (A) Expressing LITE-1 pan-neuronally is sufficient to rescue the light response of a *lite-1* null. Shown are the mean locomotion rates of each strain in low power white light (dark bars) or a low power of blue light (see Materials and Methods). Higher powers paralyze *lite-1* transgenic animals (Video S8). Error bars are the standard errors in populations of ten animals. (B) Expressing LITE-1 in cholinergic motor neurons partially rescues the light response of a *lite-1* null. Legend description for (A) applies. See also Video S9, which shows that these animals coil up and become paralyzed under higher light powers.

To determine if LITE-1 functions in the nervous system to control light-induced locomotion, we produced a transgenic strain containing the *lite-1* cDNA under control of a pan-neuronal promoter in a *lite-1* null mutant. Illuminating this strain with a power of blue-violet light that maximizes the wild-type response caused a brief acceleration followed by rapid paralysis (Video S8). However, reducing the light potency by switching to blue light, and decreasing the power to 16% of the optimal power for wild type, produced a light response in the transgenic strain that did not significantly differ from the wild-type response in initial robustness (∼4.5-fold increase in locomotion rate for both strains) (Figure 8A and Video S8). However, unlike wild type, the transgenic strain could not maintain its high locomotion rate for more than 2–3 min (NKC, KGM, unpublished data), and the data in Figure 8A represent the locomotion rate during the interval from 2–3 min after the start of light exposure. Expressing the *lite-1* cDNA only in the cholinergic motor neurons of a *lite-1* null mutant conferred light-induced coiling and paralysis at blue light powers optimal for wild type, but reducing the light power 10-fold increased its light-induced locomotion rate ∼3-fold (Figure 8B and Video S9) during the interval from 1.5–2.5 min after the start of illumination. This suggests that LITE-1 can strongly stimulate the activity of cholinergic neurons; however, it is unclear whether cholinergic neurons overlap with LITE-1′s native site-of-action, or whether LITE-1 is simply sufficient to confer light-induced activation of cholinergic neurons.

### LITE-1′s Site-of-Action for Light-Induced Forward Locomotion Is in the Tail

**Figure 9:**
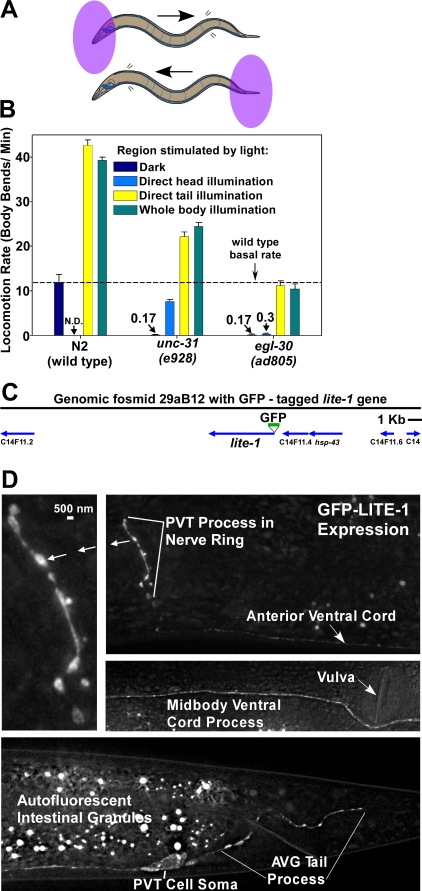
Sites of Action of LITE-1 (A) Drawings showing the areas illuminated in the regional illumination experiments. (B) Whole body or tail-only illumination rescues synaptic signaling mutants equally well. Numbers above some bars state the locomotion rates of near-paralyzed mutants. We did not assay N2 (wild type) for head illumination due to difficulty in maintaining head only illumination during the assay. Error bars are the standard errors in populations of 10 animals (whole body), 30 animals (*egl-30* – head and all tail light assays; 1 min each), or 60 animals (*unc-31* – head light assay; 30 s each). (C) Scale drawing shows the GFP-tagged genomic fosmid we used to make the GFP-LITE-1 transgenic strain *ceIs51*. Arrows show gene locations and directions of transcription. (D) The LITE-1 receptor is expressed in only two neurons and concentrates in single head and tail processes. Images are from a *lite-1* null mutant containing the *ceIs51* transgene. GFP-LITE-1 is only detectable in the soma and process of the PVT neuron as well as the tail process (but not the soma) of the AVG neuron. The blobby swellings in the head and tail may represent the animal's light sensors. See also Figure S6.

To investigate LITE-1′s site-of-action, we used a low-power blue-violet laser to specifically illuminate the head or tail of wild-type animals on culture plates (Figure 9A). The initial direction of movement was backward in 48/50 trials in response to head illumination and forward in 50/50 trials in response to tail illumination. The dominant response is forward movement, because the initial direction of movement during whole body illumination was forward in 48/50 trials. Unexpectedly, either whole-body or tail-only illumination rescued the paralyzed synaptic signaling mutants equally well, whereas head-only illumination produced only a weak response in the *unc-31* mutant and no response in the *egl-30* mutant (Figure 9B). Together with our transgenic rescue data showing a site-of-action in neurons, these data show that LITE-1′s dominant site-of-action with respect to light-induced forward locomotion is one or more tail neurons or tail neuronal processes.

Because *lite-1* is part of a very large and complex operon with widely dispersed regulatory elements (unpublished data), we were unable to define a rescuing promoter for use in driving a transcriptional GFP reporter to determine where LITE-1 is expressed. We therefore recombineered GFP onto the N terminus of the *lite-1* gene in the context of a large fosmid containing all of the presumptive regulatory sequences for *lite-1* expression (Figure 9C). The GFP-LITE-1 fosmid transgene is sufficient to rescue the light response of a *lite-1* null mutant to near wild-type levels (Figure S5). In multiple integrated lines made from this tagged fosmid, we detected GFP-LITE-1 in only two neurons, one of which we identified as PVT based on its position, its large elongated cell soma, and its process morphology. PVT produces GFP-LITE-1 in its cell body in the posterior of the animal and exports it via a ventral nerve cord process to a terminal region with large swellings in the nerve ring (in the head) where GFP-LITE-1 concentrates (Figure 9D). The other neuron has a process with large swellings of concentrated GFP-LITE-1 in the tail (Figure 9D). Although we were not able to detect the other neuron's cell body, its process belongs to AVG based on the unusual swellings, its unique route through the dorsorectal commissure, and its wavy path and abrupt termination in the mid-tail region (Zeynep Altun and David Hall, personal communication; Figure S6). Previous studies showed that AVG and PVT both contribute to establishing or maintaining ventral cord axonal tracks in embryos or newly hatched larvae [18–21]; however, their role in older larvae and adults is unknown. Since we can only detect the rescuing LITE-1 transgene in these two neurons, our data suggest that the distal processes of PVT and AVG may be sufficient to function as tail and head light sensors for light-induced forward and reverse locomotion, respectively. However, we cautiously note that laser ablation experiments, in which we individually eliminated each of these neurons, did not affect the forward or reverse light response (unpublished data). We therefore conclude that LITE-1 must function in other neurons as well, where it must be below our level of detection using the GFP-LITE-1 transgene.

### LITE-1 Is a Light Receptor

**Figure 10:**
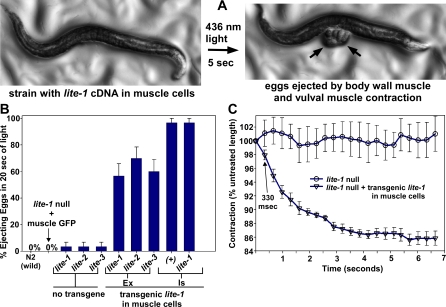
LITE-1 Is Sufficient to Confer Light Responsiveness to Muscle Cells (A) Blue-violet light-induced contraction of body wall muscles forces egg ejection in transgenic animals expressing the *lite-1* cDNA in body wall muscle. Genotype of the strain is *lite-1(ce314); ceIs37* (*myo-3::lite-1* cDNA). (B) Shown are the percent of animals that ejected eggs during a 20-s stimulus of 436 nm light. Note that the LITE-1 – induced muscle contraction does not require LITE-2 or −3. Genotypes of transgenic strains from left to right: *lite-1(ce314); ceEx190* (*myo-3::*GFP), *lite-1(ce314); ceEx186* (*myo-3::lite-1* cDNA), *lite-2(ce311); ceEx189* (*myo-3::lite-1* cDNA), *lite-3(ce360); ceEx188* (*myo-3::lite-1* cDNA), *ceIs37* (*myo-3::lite-1* cDNA), *lite-1(ce314); ceIs37* (*myo-3::lite-1* cDNA). “Ex” and “Is” denote transgenic strains with extrachromosomal and integrated arrays. Error bars are the standard errors in populations of 30 animals. (C) Time course of LITE-1-mediated muscle contraction. Genotypes are *lite-1(ce314)* and *lite-1(ce314); ceIs37* (*myo-3::lite-1* cDNA). Data represent the mean and standard errors of five animals each.

Given that LITE-1 is concentrated in regions that sense light, and that it has homology to receptors, we hypothesized that it could be a novel ultraviolet light receptor, and we obtained compelling evidence for this. Expressing *lite-1* heterologously in body wall and egg laying muscles conferred light responsiveness to a tissue that is normally unresponsive to light. In these transgenic animals, blue-violet light caused a rapid and powerful muscle contraction and shortening of body length. This, combined with activation of the egg laying muscles, caused egg ejection as the increased internal pressure caused by the body contraction forced eggs out of the now open vulva (Video S7). The light-induced egg ejection only occurred in strains containing the *lite-1* cDNA in muscle cells and not in any of the control strains (Figure 10B). By imaging animals as they contracted in the light, and measuring their lengths at each time point, we found that light exposure induces body contraction within 330 ms, and the contraction is complete by 3–4 s (Figure 10C). Interestingly, the light-induced contraction was undiminished in *lite-2* or *lite-3* mutant backgrounds. Since the *lite-2* and *lite-3* mutants have Lite phenotypes as strong as *lite-1* nulls (NKC, KGM, unpublished data), these data suggest that LITE-1 can function independently in heterologous cells as a light receptor.

## Discussion

Here we report a novel sensory modality in C. elegans: photosensation of ultraviolet light. The fact that C. elegans has a robust UV light response suggests that it often lives on surfaces that could be exposed to direct sunlight. In support of this, a recent study failed to find C. elegans in soil, but instead found it on snails [5]. Other studies have found C. elegans on terrestrial isopods, millipedes and other arthropods, and slugs [6]. According to one theory, dauer juveniles embark on an animal and wait for it to die. They then resume development and propagate on the decomposing body [6]. Interestingly, in quantitative assays, dauer larvae have an exceptionally strong light response. For example, the levels of blue-violet light that maximize the responses of wild-type and *unc-31* mutant adults, which result in 3.5-fold and 65-fold locomotion rate increases, respectively, cause 20-fold and 1,400-fold locomotion rate increases in dauer larvae from the same strains (NKC, KGM, unpublished data). Having such a robust mechanism for escaping damaging doses of short wavelength light would allow C. elegans to avoid the potentially lethal doses of sunlight that may often permeate its above ground habitat.

By using forward genetic screens, we discovered a novel molecular solution for ultraviolet light detection that is evolutionarily tailored to activate neurons. LITE-1 has no homology to any of the six known photoreceptor families: rhodopsins, phytochromes, xanthopsins, cryptochromes, phototropins, and BLUF proteins [22]. Each of these families associates with one or more small-molecule chromophores, such as retinal or flavin-based molecules that interact with photons to activate the protein [23–26]. Since LITE-1 has none of the known chromophore interacting domains or residues, it is not clear whether it has a permanently bound chromophore, or whether it binds to a photo-oxidation product produced by short wavelength light. LITE-1′s homology to gustatory receptors that bind small, water-soluble molecules is consistent with either possibility.

LITE-1 is one of only several *Gr* family members for which a loss-of-function phenotype has been described. In *Drosophila*, recent studies have shown that *Gr64a* and *Gr5a* mediate the sensation of most or all types of sugars [27], and that *Gr21a* and *Gr63a* together mediate CO_2_ (or bicarbonate ion) reception [28–30]. Our addition of ultraviolet light to this emerging list is unexpected. LITE-1′s membership in the *Gr* family provides no clues about how the receptor exerts its effects. The mechanism of action of *Gr* receptors remains unknown, largely because they have been notoriously difficult to express in heterologous systems. LITE-1 is no exception to this (unpublished data). Although similar in membrane topology to G protein–coupled seven transmembrane receptors, the *Gr* family is unrelated to G protein–coupled receptors at the sequence level, and there is no direct evidence that *Gr* receptors exert their effects through G proteins.

Perhaps the most interesting aspect of the C. elegans light response is the light-induced rescue of near-paralyzed synaptic signaling mutants, and our finding that tail illumination is sufficient for this rescue. These findings show that a small subset of neurons, possibly as few as one based on our rescuing transgene analysis, can drive a robust, coordinated locomotion response that largely bypasses the synaptic signals that are required for locomotion under normal lighting. A recent study found that mice neurons lacking both CAPS-1 and CAPS-2 (the mouse orthologs of UNC-31) are strongly defective in synaptic vesicle priming and neurotransmitter release when electrically stimulated for short periods of time, but that this defect can be rescued by the large Ca^++^ increases that accompany extended trains of electrical stimuli [31]. Our finding that LITE-1 induces a powerful light-dependent contraction of muscle cells is consistent with LITE-1 activity ultimately increasing calcium levels in excitable cells. We hypothesize that chronic firing of light circuit neurons during exposure to light could raise internal Ca^++^ to the level necessary to bypass the need for the Gα pathways. The ∼10-s delay in the response of the *unc-31* and Gα_q_ mutants to light stimulation versus 1–2 s for wild type seems consistent with the time that would be needed for the light-induced buildup of a stimulatory signal at each sequential synapse in a polysynaptic circuit stretching from sensory neurons through interneurons and, ultimately, to the motor neurons and the muscle cell. Future studies may be able to harness LITE-1 as a tool for the photoactivation of neurons in living animals and cells or for the investigation of cAMP- and DAG-based synaptic signaling pathways in both invertebrate and vertebrate systems.

## Materials and Methods

### Worm culture and manipulation.

See Text S1 for specialized worm culture methods. Locomotion plates were made as described [32]. Other worm culture and manipulation essentially followed previously described methods [33,34]. We prepared 24-well culture plates for genetic screens as previously described [9].

###  Strains.

Wild-type strains were N2 (Bristol) [33] and CB4856 (Hawaiian) [35] as indicated. During outcrossing of *tax-2(p691)*, we discovered that the original strain contained a second site mutation in *lite-3*, which we designated *ce360*. Text S1 gives a complete strain list for this study.

###  Laser light source.

We used a 405-nm, 5-mW laser with 0.9-mm-diameter beam size (CrystaLaser #BCL-005–405) with CL2005 adjustable power supply. The laser head was mounted to a standard laboratory stand using a standard laboratory clamp. We positioned the laser to project onto the culture plate as near to vertical as possible in the center of the field of view while viewing animals through an Olympus SZX-12 stereomicroscope equipped with a 1.2×, 0.13 numerical aperture plan apochromatic objective.

###  Mercury light source and light measurements.

We used the equipment shown and described in Figure S7 to project light of the desired wavelength and power through the stereomicroscope objective onto the culture plate surface. We used the standard Chroma filters shown in Figure 1B to restrict wavelengths to various ranges from UV to red. To measure the light power per mm^2^ at the culture plate surface, we always placed the detector in a defined location/orientation on the microscope stage due to inherent variation of different regions of the detector surface. We then zoomed to 108× to concentrate a light beam of 9.62 mm^2^ onto a subregion of the detector.

###  Locomotion assays.

Text S1 describe specialized procedures for light-dark locomotion assays, regional illumination locomotion assays, and short– and long–time course locomotion assays.

###  Temperature experiments.

Text S1 provides detailed descriptions of methods for measuring the temperature changes induced by direct illumination of a Checktemp 1 digital temperature probe (Hanna Instruments), methods for measuring the light-induced temperature changes of worm pellets, and methods for testing the effects of temperature on the locomotion rate of the *unc-31* null mutant.

###  Crowded plate light avoidance assays.

We produced synchronous populations of unstarved young adults on spread plates (4,500 per plate × 10 plates) and adjusted the light source color and power as described above and in Figure S7. After putting the first plate on the scope stage under low intensity white illumination, we focused on a random area near the center of the plate, and zoomed up to 108×. We then switched the light path to green or blue-violet light (1,460 μW/mm^2^) and simultaneously started both channels of a timer counting down at 45 s and 5 min 45 s. At 45 s, we counted the number of adults that were fully in the field of view through eyepieces. Over the next 5 min, we then counted the number of animals that subsequently fully entered the field of view. If an animal that was in the field of view partially left the field of view and then fully re-entered, we counted it.

###  Sunlight power measurements.

We obtained 32-mm-diameter excitation filters from Chroma (D350/50x for ultraviolet; D436/20x for blue-violet; and HQ470/40x for blue). These excitation filters have identical properties to the excitation filters used in the nosepiece of the stereomicroscope to produce colored light on the culture plates. Each filter has a metal frame that fits precisely over the Newport 818-UV detector with the 1,000× attenuator attached (Figure S7). We sealed around the interface of the detector and filter with opaque tape to prevent light leakage. We used the Newport 830-C power meter (see Figure S7), set on the center wavelength of each filter, to take the readings. The readings were taken at solar noon ± 10 min on February 4, 2006, in Edmond, Oklahoma, in full sunlight outdoors. To take readings, we pointed the detector with its filter attached directly at the sun and noted the maximal reading that occurred when the angle was optimal. We then adjusted this reading for the percent transmittance of each filter (55% for D350, 70% for D436, and 73% for HQ470) and normalized the readings to power per mm^2^, based on the detector surface area as stated by the manufacturer (100 mm^2^).

###  Genetic screen for Lite mutants.

To isolate Lite mutants, we first produced and plated ∼30 adult F2 grandprogeny of EMS-mutagenized N2 (wild type) in each well of 24-well culture plates. We used two methods to screen wells for Lite mutants. For Method 1, we used a 405-nm, 5-mW laser with 0.9-mm-diameter beam size (CrystaLaser; #BCL-005–405) with a CL2005 adjustable power supply. We mounted the laser head to a standard laboratory stand using a standard laboratory clamp. We positioned the laser to project onto the culture plate as near to vertical as possible in the center of the field of view while viewing animals through an Olympus SZX-12 stereomicroscope equipped with a 1.2×, 0.13 numerical aperture (NA) plan apochromatic objective. We set the laser power on 5.0 mW. We kept the laser in a fixed position and moved the plate to illuminate the desired region. To screen for Lite mutants, we simply positioned a 24-well plate on the scope stage and, starting with the first well, moved the plate such that an adult animal was illuminated over most of its body by the laser light. If the animal didn't move away within 3 s or so (usually less), we marked its position (using a pick mark in the bacterial paste), picked it to a streak plate for further observation, returned to the marked position, and then moved on to test the next animal, etc. and proceeded systematically to test each adult animal in the well before moving on to the next well. For Method 2, we screened the wells using a mercury light source and the CFP filter. The light power was ∼1,500 μW/mm^2^ at the screening magnification of 108×. We moved animals into the field of view by moving the plate and noted their light responses (single or multiple animals at a time). We used Methods 1 and 2 about equally. We picked candidate Lite mutants all to the same streak plate for further testing of their light responses. We discarded paralyzed or very sluggish animals, and cloned putative Lite mutants to individual streak plates. After growing 4–5 d at 20 °C to produce adult progeny of the original mutants, we discarded obvious non-Lite mutants, subjectively scored real Lite mutants for the strength of their light responses, and confirmed the homozygosity of the strain or cloned candidate homozygotes if, as in several cases, the original mutant was heterozygous. We carried out the screen in 13 consecutive weekly cycles of screening 4 d/wk with 3–5 people screening ∼2 h/d to achieve the screening goal of 250,000 F2s.

### *lite-1* mutant complementation testing and outcrossing.

We outcrossed most Lite mutants at least twice by crossing *lite/+* males with *dpy-5(e61)* hermaphrodites and then re-isolating Lite animals in the F2 generation. To make the 5× outcrossed *lite-1* null mutant reference allele *ce314*, we first outcrossed it once through N2 and then repeated the above *dpy-5* crossing procedure twice. For some mutants, including *ce314*, we also isolated the *dpy-5(e61); lite-1* double mutant from the progeny of this cross for use as a marked strain for complementation testing. We complement tested all Lite mutants by crossing Lite/+ males with *dpy-5; lite-1(ce302)* or *dpy-5; lite-1(ce314)* and scoring adult non-Dpy cross progeny for their Lite responses.

###  SNP mapping of Lite mutants.

During outcrossing, we found that all of the Lite mutants are X-linked. To map *lite-1(ce302)* and *lite-1(ce314)* to a subregion of the X chromosome, we crossed CB4856 males to *lite-1* mutant hermaphrodites and re-isolated putative *lite-1* homozygotes in the F2 generation of this cross. We then checked the adult progeny of these animals for homozygosity (absence of wild-type animals), and, upon starvation, we checked the homozygous cultures for various X-linked CB4856 SNPs as described [9]. We used snip-SNPs identified by [35] to map the mutations to a subregion in a manner similar to that previously described [9]. We combined the mapping data for *ce302* and *ce314* based on our noncomplementation data. After using snip-SNPs to identify recombinants that break left and right of the *lite-1* locus (between *ceP173* and *ceP171*), we tested the recombinants for the presence of other SNPs in the *ceP173* – *ceP171* interval by restriction analysis or sequencing as described [9]. The SNPs that we used to narrow *ce302* and *ce314* to the final interval have been previously reported as locations on specific genomic DNA clones [35,36]. The specific locations are as follows (genomic DNA clone/ location on clone; in order from left to right on chromosome) : *ceP173* (Y23B4A/ 12,491); *ceP43* (T13C5/ 12,745); *ceP176* (T10E10/ 6753); *ceP179* (T22E5/ 27,633); *ceP171* (F38B6/ 13,786).

###  Sequencing *lite-1* mutations.

After identifying the *ce302* and *ce314* mutations from the above analysis, we sequenced genomic DNA from the other 16 *lite-1* mutants by making crude plate lysates from a freshly starved streak plate of each strain. We then amplified the *lite-1* gene from the lysates using Expand 20 Kb+ and sequenced the resulting products.

###  Double mutants.

We constructed the *egl-30(ad805); lite-1(ce314)* and *unc-31(e928); lite-1(ce314)* double mutants using standard genetic crossing methods.

###  DNA constructs and transgenes.

Text S1 describes all of the DNA constructs and transgenes used in this study. In all constructs involving the cloning of PCR fragments, we sequenced the inserts and used clones containing no mutations in the fragment of interest to establish the final plasmid stock. We produced transgenic strains bearing extrachromosomal arrays by the method of Mello et al. [38]. We used pBluescript carrier DNA to bring the final concentration of DNA in all injection mixtures to 175 ng/μl. All injection mixtures in this study included the co-transformation marker plasmids containing the same promoter as the experimental DNA, but hooked to GFP instead. The injection mixtures, plasmid concentrations, and host strains of all of the transgenic strains used in this study are listed in Text S1. We produced integrated arrays using previously a described method [10], except we screened cultures for 100% transmittance of GFP, and we used 7,200 Rads of γ irradiation. We outcrossed *ceIs37* twice to wild type, keeping versions with and without *lite-1(ce314)*. We confirmed the homozygous presence and absence of the *ce314* mutation by PCR and sequencing.

###  Recombineering.

We recombineered GFP onto the N terminus of the *lite-1* gene on the fosmid WRM062dF04 (Geneservice) to make the new fosmid KG#319 using a modification of a previously described method [37]. Briefly, we substituted the plasmid pRedFlp4 (gift of Mihail Sarov) for pRedFlp as a source of the Red/ ET recombination proteins and Flp recombinase. pRedFlp4 substitutes the HgrR hygromycin resistance gene for the AmpR gene in pRedFlp. Whereas the original protocol required Amp/ Trimethoprim to select for pRedFlp transformation into the fosmid host, the modified method uses only Hygromycin (we used HygroGold (InvivoGen at 200 μg/ ml)). Trimethoprim cannot be used for transforming into fosmid hosts since fosmids contain the DHFR gene that confers Trimethoprim resistance. In addition, we modified the pR6KGFP plasmid to allow N-terminal or internal fusions to the gene of interest. The new plasmid (pR6KGFPX) lacks a GFP stop codon, contains a two-nucleotide “GG” insertion immediately after the 34-bp FRT and before the reverse primer homology region to maintain reading frame with the downstream protein, and changes an in-frame TGA stop codon in the reverse primer homology region to GGA. Finally, we did not subclone the GFP-tagged *lite-1* gene from the fosmid into the pPUB vector but instead left it as a GFP-tagged fosmid. The final tagged-product structure is as follows: ATG of *lite-1* gene → 6 forward primer codons → ATG of GFP → GFP coding sequences minus stop codon → 1 copy of FRT → GG nucleotides → reverse primer codons → the rest of the *lite-1* gene and genomic sequences on the fosmid. We confirmed the final fosmid structure by PCR to detect insertion of GFP and to confirm that no fosmid lacking GFP was present in the clone, and we sequenced the insertion region.

###  Egg ejection and muscle contraction assays.

For the egg ejection assay, we picked 30 gravid adults from growing cultures to a standard spread plate, spacing them in different regions of the plate (see Text S1 for “growing cultures” and “spread plate” definitions). Each adult carried ≥ 6 eggs. If the strain contained a GFP-marked extrachromosomal array, we required that the adults show uniform green in their muscle cells. To avoid making the GFP-positive animals eject their eggs while choosing them, we zoomed out as far as possible when the GFP light was on (to reduce its intensity) and worked quickly. As soon as we identified a uniform green, GFP-positive animal, we switched to standard white light to examine egg number and to make the transfer. With no plate on the stage, we adjusted the total CFP light power to 14.6 mW at 108× magnification, and then switched back to normal white light. We removed the lid from the plate containing the 30 animals to be assayed, focused on the first animal, zoomed to 108× magnification, and centered the field of view on the animal's vulva. We then turned up the white stage light such that we would be able to clearly see the animal after switching to CFP light, and then simultaneously started the timer and slid the CFP filter into place. At the end of the 20 s, we switched the light back to white stage light and noted whether any eggs were dumped during the 20-s stimulus. We picked off the animal we had just assayed, re-checked/ adjusted the light power, and repeated the assay on the other 29 animals for each strain.

To take the time course of light-induced muscle contraction, we produced growing cultures of each strain on spread plates. Under normal white stage light illumination, we transferred five gravid adults to a standard spread plate for each strain. We required that each adult be carrying ≥ 6 eggs. With no plate on the stage, we adjusted the CFP light power to 32.5 mW total power at 108× magnification. We then increased the white stage light as far as possible to shorten exposure time which, under these conditions, was ∼50 ms. After choosing the first animal to assay on the culture plate (plate lid removed), we zoomed to 75× and centered the animal, which took up ∼75% of the camera screen field of view. We then simultaneously switched to the CFP light path and clicked the “Acquire” button to collect a 10-s time course of images, spaced 330 ms apart, using an ORCA-AG camera and Metamorph Premier software (Version 6.3 r1). We used the multi-line region tool and clicked along the midline of each animal from the tip of the nose to a defined point near the end of the tail. We logged length measurements in units of pixels and used an Excel spreadsheet to convert data to the final units of “percent unstimulated length” for each time point.

###  Imaging.

We collected fluorescent images using a Nikon Eclipse TE2000-E inverted microscope equipped with a 60× 1.4 N.A. oil planapochromat objective (CF160-type), a 1.5× tube lens, a motorized linear-encoded z-drive, and a motorized filter turret containing a Semrock GFP filter cubes. Our illumination source was an X-Cite 120 illuminator (EXFO), and we captured 12-bit images with an ORCA-AG camera (Hamamatsu) controlled by Metamorph Premier software (Version 6.3 r1). We further processed z-series stacks of images by the Adaptive PSF Blind Deconvolution method (10 iterations with low noise level setting) using AutoDeblur Gold CWF software (ImageQuant). We then used AutoDeblur to resize the stacks 2-fold in the X and Y dimensions, and then used Metamorph to produce maximum projections of the stacks, adjust the scaling and generate 8-bit images for display.

## Supporting Information

Figure S1 The Synaptic Signaling Pathways that Drive Locomotion in C. elegans
Shown is a model of the three major Gα pathways (Gα_q_, Gα_s_, and Gα_o_) that control locomotion rate in C. elegans. Solid lines indicate that direct interactions are known or likely, while dashed lines and/ or large gaps between line endpoints and downstream effectors indicate predicted interactions or predicted missing components. Proteins that promote locomotion and/or neurotransmitter release are shown in green, while proteins that inhibit locomotion and/ or neurotransmitter release are shown in red. Asterisks indicate proteins that were tested for a role in the light response using the mutants described in Figure 1. See text for a brief summary and the following references for details [8–10,32,39–60].
(70 KB PDF)

Figure S2 The Wild-Type Response to Light Is Restricted to Short Wavelengths Shown are the mean locomotion rates, expressed as body bends per min, of wild type illuminated with various wavelengths at a constant power. Dashed lines indicate basal locomotion rates in low power white light and in the highest power of UV light that we could project onto the culture plate. Although the UV light is only one-seventh of the power of the other wavelengths, it produces a response similar to a much higher power of blue-violet light. Error bars are the standard errors of 10 animals.
(48 KB PDF)

Figure S3 Death in Blue Light Is Not Caused by Overactivation of LITE-1 Extended time course assay comparing N2 (wild type) and *lite-1(ce314)* using near-maximum power of blue-violet light from a standard mercury light source. Shown are the mean locomotion rates at various times during exposure to four times the optimal power of blue-violet light. Following a vigorous locomotory response to high power blue-violet light, wild type rapidly slows its locomotion on the culture plate, and then dies after ∼25 min of exposure. A *lite-1* null mutant lacks the initial response to light, but becomes damaged in light and dies with a time course that is indistinguishable from wild type. Error bars are the standard errors of four animals. Light power was 2,800 μW/mm^2^ using the CFP filter (blue-violet, 436 nm). N2 data are from Figure 4D.
(37 KB PDF)

Figure S4 LITE-1 Is a Predicted Eight-Transmembrane Protein with Homology to *Drosophila* Gustatory Receptors Shown is an amino acid alignment of C. elegans LITE-1 (Ce LITE-1), C. briggsae LITE-1 (Cb LITE-1), and D. melanogaster Gr28b (Dm Gr28b) based on the Clustal W algorithm [61]. The TMHMM algorithm [62] predicted the transmembrane domains (boxed) and that the N and C termini are on the outside of the cell membrane. We obtained similar results using the HMMTOP algorithm [63,64], although the exact boundaries of the predicted transmembrane domains differed between TMHMM and HMMTOP. Arrows indicated the locations of all of our LITE-1 missense mutations.
(236 KB PDF)

Figure S5 Expression of GFP-LITE-1 from the *ceIs51* Transgene Is Sufficient to Restore Light-Responsiveness to a *lite-1* Null Mutant (A) A *lite-1* null mutant containing the *ceIs51* transgene that expresses GFP-LITE-1 in AVG and PVT restores near wild type light-responsiveness. Error bars are standard errors. *n* = 20 animals each in 2-min locomotion assays following a 30-s equilibration to the light.
(B) Expression of GFP-LITE-1 from the *ceIs51* transgene confers near normal forward and reverse responses to tail and head illumination. Tables show the number of animals showing the indicated initial response after the indicated head or tail illumination stimulus. *n* ≥ 50 per strain per stimulus.
(52 KB PDF)

Figure S6 The Tail Process in the GFP-Marked Rescuing Transgene Is the AVG Process (A) A LITE-1 positive process passes near and sometimes over the PVT cell soma on its way to the tail. Two images from a *lite-1(ce314)* mutant rescued with the *ceIs51* transgene (*lite-1::GFP-lite-1* on a fosmid).
(B) The LITE-1 tail process has a distinct beaded appearance, often ending abruptly in the mid-tail with a swelling. In this image from *lite-1(ce314); ceIs51* (*lite-1::GFP-lite-1*), the tail process appears to be coming from PVT; however, PVT does not send a process into the tail, and some images clearly show a process running over PVT [e.g.,(A)].
(C) The AVG tail process resembles the LITE-1 – positive tail process. In this image from *akIs1* (nmr-1::GFP), a beaded AVG tail process ends abruptly in the mid-tail with a swelling similar to the LITE-1–positive process. This is a transcriptional fusion of the *nmr-1* promoter driving cytoplasmic GFP [65]. This promoter also expresses in the PVC neurons in the tail, one of which is visible in the image.
(302 KB PDF)

Figure S7 Method for Measuring and Controlling Light Power through a Stereomicroscope Objective Our light source is a 120-W mercury bulb in an X-Cite 120 Illuminator (EXFO). White light from this bulb travels to the microscope via a liquid light guide. We control the power of the light leaving the illuminator with the click stop iris on the X-Cite illuminator (often nudging the iris to intermediate positions to achieve the desired power). The white light passes through an excitation filter in the nosepiece that only allows certain wavelengths to pass through the objective. We measure the power of light reaching the culture plate with a Newport 818-UV detector connected to a Newport 830-C power meter that is calibrated for all of wavelengths from UV to infrared. The detector has 1000× attenuator screwed onto it for measuring high power light readings, and the power meter is set in “attenuator” mode and adjusted for the wavelength being measured. Markings on the microscope stage glass (not shown) allow precise alignment of the light detector such that power readings of the light beam, which is smaller than the detector area, are always taken from the same region of the detector. Note: the maximum power output of the X-Cite Illuminator can vary significantly from day to day, due to a phenomenon that the manufacturer calls “arc wander”. For example, the blue-violet maximum power varied from ∼2.4–∼3.7 mW/mm^2^. This and other factors such as bulb and liquid light guide age and condition also affected the maximum power we could project onto a culture plate and sometimes affected the timing of our experiments (i.e., to obtain data for high power light for the dose-response curves).
(87 KB PDF)

Figure S8 Loss-of-Function Mutations in the TAX-2 and TAX-4 CNG Channels Do Not Affect the Overall Locomotion Response to Short-Wavelength Light Shown are the mean locomotion rates of wild type and the indicated CNG channel mutants in 70 nW/mm^2^ white light (dark bars) or blue-violet light that maximizes the wild-type response (730 μW/mm^2^; light bars). The locomotion rates reflect both forward and reverse locomotion, although forward locomotion dominates during whole-body illumination, as shown in Figure 9. Note that *tax-2(p671)* appears to have a small reduction in its locomotion rate in blue-violet light relative wild type; however, its fold-increase from its basal rate (4.15-fold) is actually greater than that of wild type (3.33-fold). Strains names are as follows: KG1352 *tax-2(p691)*, PR671 *tax-2(p671)*, FK108 *tax-4(ks28)*, and PR678 *tax-4(p678)*. Error bars are the standard errors in populations of ten animals.
(28 KB PDF)

Table S1 Representative Examples of Light Power (19 KB PDF)

Table S2 *lite-1* Mutations (15 KB PDF)

Text S1 Supplementary Methods Includes specialized procedures for worm culture for various assays in this study, complete lists and descriptions of strains, plasmids, and transgenes, specialized assays for locomotion rate during light exposure, and methods for the temperature experiments.
(99 KB PDF)

Video S1 An *unc-31* Null Fails to Respond to Harsh Poking with a Metal Pick Illumination is diffuse white light. The animal is on a lawn of bacteria, which is its food source.
(2.03 MB MOV)

Video S2 Blue Light Induces Coordinated, Wild-Type Locomotion in an *unc-31* Null Video begins with slamming the plate down hard against the scope stage several times to show that these mutants are unresponsive to harsh physical stimuli. It then zooms up to 108× magnification so the blue light will be concentrated on the animal. A brief darkening indicates the switch to blue light (from a GFP filter), which remains on for the rest of the movie. The white stage light is then turned up at the same time so viewers can see the animal. The animal is on a lawn of bacteria, which is its food source.
(8.62 MB MOV)

Video S3 A Gα_q_ Mutant Fails to Respond to Harsh Poking with a Metal Pick Illumination is diffuse white light. Genotype is *egl-30(805)*.
(1.29 MB MOV)

Video S4 Blue Light Induces Coordinated, Wild-Type Locomotion in a Gα_q_ Mutant Video begins with slamming the plate down hard against the scope stage several times to show that these mutants are unresponsive to harsh physical stimuli. It then zooms up to 108× magnification so the blue light will be concentrated on the animal. A brief darkening indicates the switch to blue light (from a GFP filter), which remains on for the rest of the movie. The white stage light is then turned up at the same time so viewers can see the animal. Genotype is *egl-30(805)*.
(6.96 MB MOV)

Video S5 Wild-Type (N2) Worms Scatter in Blue Light The movie starts with a population of animals foraging on a lawn of bacteria in standard low power white light. The brief darkening ∼15 s into the movie indicates the switch to blue light, which remains on for the rest of the movie. Note that the animals rapidly scatter from the field of view during blue light exposure.
(2.57 MB MOV)

Video S6 *lite-1(ce302)* Worms Do Not Scatter in Blue Light The movie is similar to the Video S5, but the blue-violet light doesn't come on until ∼half way through the movie as indicated by the brief darkening at ∼30 s. The blue-violet illumination is of a power that maximizes the light response in both wild type and *unc-31(e928)*. Note that the animal's locomotion does not obviously change in response to blue-violet light. Near the end of the movie, the animal is stimulated with a metal pick to show that it responds normally to physical stimulation.
(4.25 MB MOV)

Video S7 LITE-1 Confers Light Sensitivity to Muscle Tissue This is a transgenic strain that expresses the *lite-1* cDNA in body wall muscle in a *lite-1* null mutant background. This 10-s video begins with illumination of the strain with blue-violet light from a CFP filter. Note the rapid contraction and egg dumping during the first 5 s of illumination. The genotype of the strain is *lite-1(ce314); ceIs37* (*myo-3::lite-1* cDNA).
(595 KB MOV)

Video S8 Expressing LITE-1 in all Neurons Is Sufficient to Rescue the Light Response of a *lite-1* Null Mutant This transgenic strain contains the *lite-1* cDNA driven by the *rab-3* neuronal promoter in a *lite-1* null mutant background. A brief darkening indicates the switch to low power blue light (GFP filter). After a brief delay the animal rapidly accelerates. At the end of the video, we rapidly zoom in on the animal to increase the light power, and the animal quickly becomes paralyzed. The genotype of the strain is *lite-1(ce314); ceEx182* (*rab-3::lite-1* cDNA).
(4.21 MB MOV)

Video S9 A Transgenic Strain Expressing LITE-1 in Cholinergic Motor Neurons Undergoes Rapid Coiling and Paralysis at High Light Powers This transgenic strain contains the *lite-1* cDNA driven by the *unc-17* cholinergic promoter in a *lite-1* null mutant background. This 30-s video begins with low power blue light illumination of the animal, which induces a loopy uncoordinated response. After ∼15 s, we rapidly zoom in on the animal to increase the light power, and the animal becomes more loopy, and then quickly coils and becomes paralyzed. The genotype of the strain is *lite-1(ce314); ceEx187* (*unc-17::lite-1* cDNA).
(2.26 MB MOV)

## Abbreviations

cAMP: cyclic adenosine monophosphate
cDNA: complimentary DNA
DAG: diacylglycerol
EMS: ethyl methane sulfonate
*Gr,*: Gustatory receptor
UV: ultraviolet

## Note Added in Proof

While this paper was in press, Ward et al. (Nature Neuroscience, 2008; doi:10.1038/nn.2155) published a paper describing the C. elegans ultraviolet light response and reporting that the *tax-2* mutants *p671* and *p691*, which carry loss-of-function mutations in a cyclic nucleotide gated ion channel, are defective in the reversal response to head illumination with UV light. In quantitative assays identical to those described here, we found that the *tax-2(p671)* and *tax-2(p691)* mutations do not affect the forward response to short-wavelength light that predominates during whole-body or tail-only illumination (Figure S8). The *lite-1* mutations described here disrupt both the forward and reverse response to short-wavelength light. For readers interested in further studying the role of TAX-2 in the reversal response to light, we note that PR691, an un-outcrossed *tax-2(p691)* strain from the C. elegans Genetics Center, has a second unlinked mutation in *lite-3*. We have separated the two mutations to produce the 4X outcrossed strains KG1352 *tax-2(p691)* and KG1214 *lite-3(ce360)*. *lite-3* maps close to *lite-1* on the X chromosome, and its light response resembles that of *lite-1* null mutants. The un-outcrossed strain PR671 *tax-2(p671)* lacks the *lite-3(ce360)* mutation.
