# Supplementary material for: A Novel Molecular Solution for Ultraviolet Light Detection in Caenorhabditis elegans
Source: PLoS Biol. 2008 Aug 5;6(8):e198. doi: 10.1371/journal.pbio.0060198 (PMC2494560; doi:10.1371/journal.pbio.0060198)
Supplement: Supplementary file 4 [file pbio.0060198.sg004.pdf]

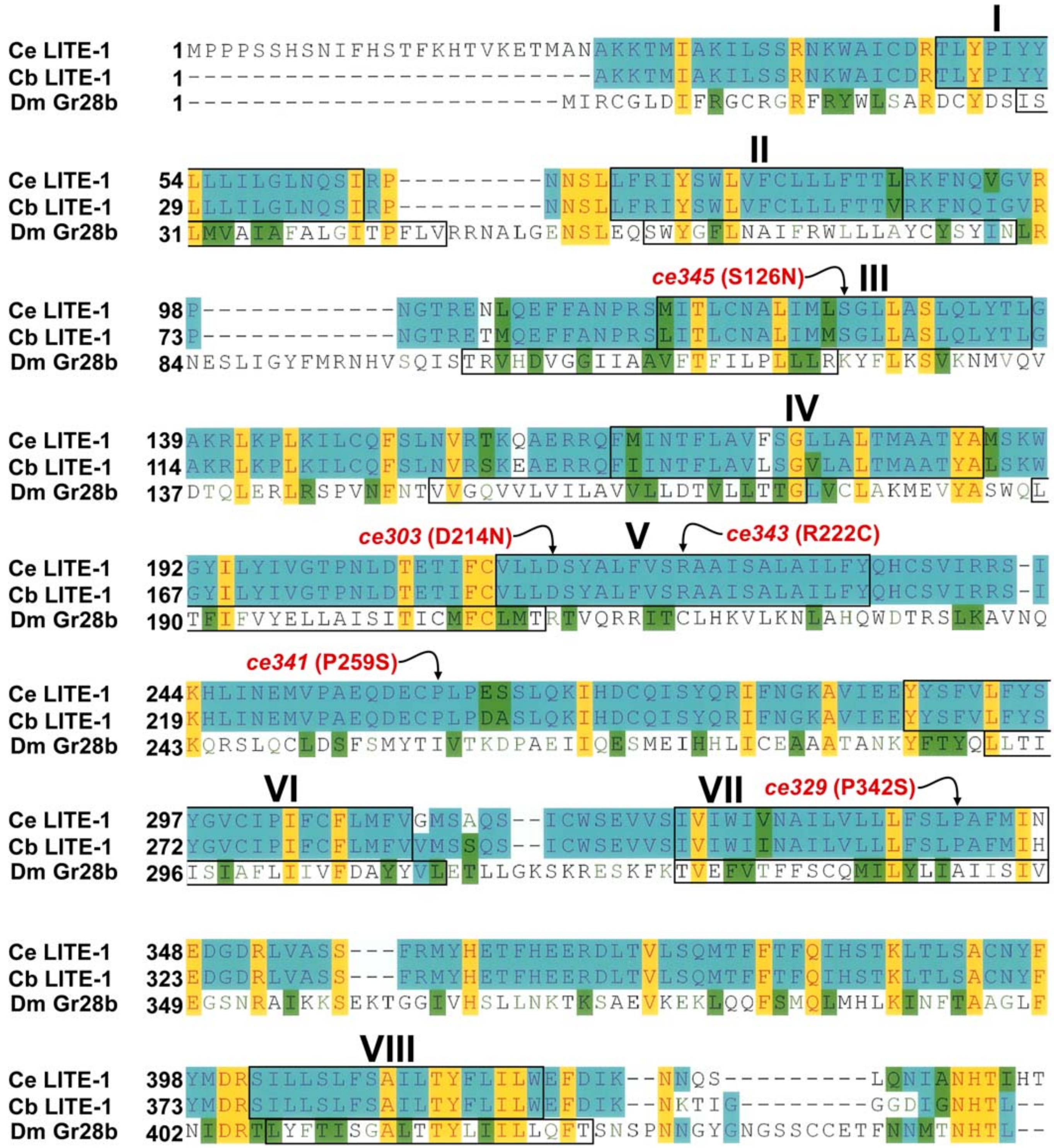

Figure S4. LITE-1 is a predicted eight – transmembrane protein with homology to *Drosophila* gustatory receptors.
