# Supplementary material for: A Novel Molecular Solution for Ultraviolet Light Detection in Caenorhabditis elegans
Source: PLoS Biol. 2008 Aug 5;6(8):e198. doi: 10.1371/journal.pbio.0060198 (PMC2494560; doi:10.1371/journal.pbio.0060198)
Supplement: Supplementary file 5 [file pbio.0060198.sg005.pdf]

A

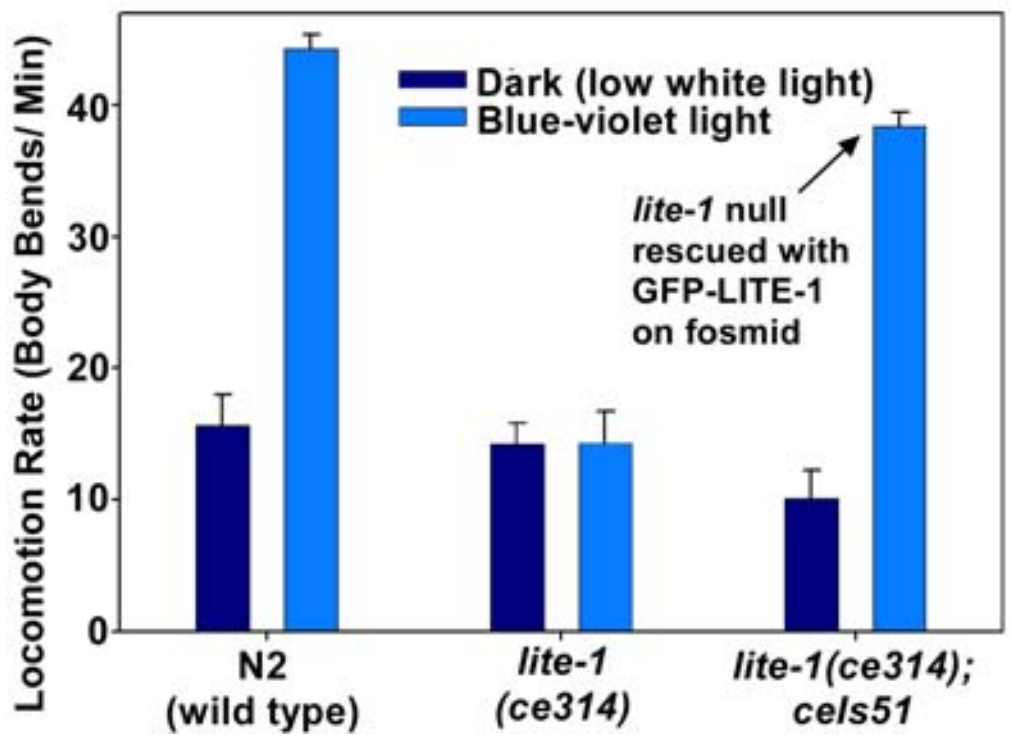


B

2 Sec Laser Head Stimulus:

| Genotype | Initial Response: Forward | Reverse | None |
|---|---|---|---|
| N2 (wild type) | 0 | 48 | 4 |
| *lite-1(ce314)* | 0 | 9 | 43 |
| *lite-1(ce314); ceIs51* | 0 | 38 | 16 |

2 Sec Laser Tail Stimulus:

| Genotype | Initial Response: Forward | Reverse | None |
|---|---|---|---|
| N2 (wild type) | 52 | 0 | 0 |
| *lite-1(ce314)* | 0 | 2 | 50 |
| *lite-1(ce314); ceIs51* | 50 | 0 | 3 |

**Figure S5. Expression of GFP-LITE-1 from the *ceIs51* transgene is sufficient to restore light-responsiveness to a *lite-1* null mutant.**
