# Supplementary material for: A Novel Molecular Solution for Ultraviolet Light Detection in Caenorhabditis elegans
Source: PLoS Biol. 2008 Aug 5;6(8):e198. doi: 10.1371/journal.pbio.0060198 (PMC2494560; doi:10.1371/journal.pbio.0060198)
Supplement: Supplementary file 8 [file pbio.0060198.sg008.pdf]

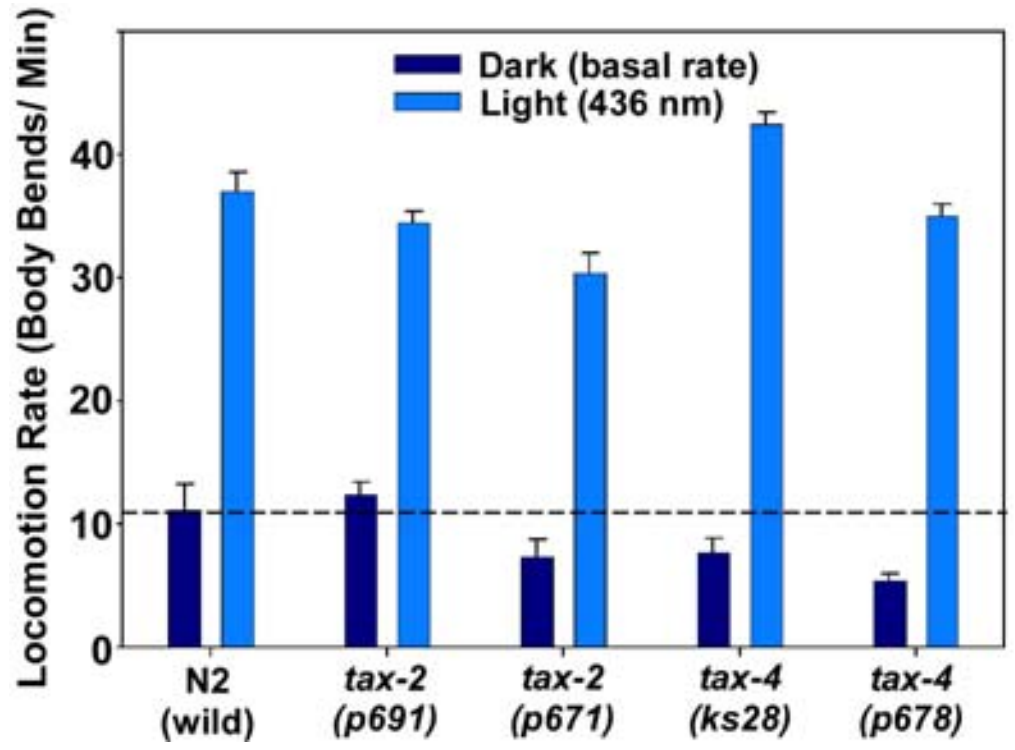

Figure S8. Loss-of-function mutations in the TAX-2 and TAX-4 CNG channels do not affect the overall locomotion response to short wavelength light
