# Supplementary material for: A Novel Molecular Solution for Ultraviolet Light Detection in Caenorhabditis elegans
Source: PLoS Biol. 2008 Aug 5;6(8):e198. doi: 10.1371/journal.pbio.0060198 (PMC2494560; doi:10.1371/journal.pbio.0060198)
Supplement: Supplementary file 9 [file pbio.0060198.st001.pdf]

Supplementary Table 1. Representative examples of light power	
**Light source**	**Light power**
[1]Stereomicroscope stage light – “dark” or “low-power white light”	0.05 μW/ mm^2
[2]Stereomicroscope stage light – Standard viewing	0.33 μW/ mm^2
[3]Ambient fluorescent room lighting	0.45 – 0.75 μW/ mm^2
[4]Minimum power of blue-violet light needed to induce the *C. elegans* locomotion response	50 μW/ mm^2
[5]Direct sunlight	1270 μW/ mm^2

For all light measurements, we set the power meter at 555 nm, which is near the center wavelength for white light. We allowed the light to fill the detector, which has an area of 100 mm^2. Values are then adjusted to power/ mm^2. For measurements of stereomicroscope light power, the detector was inverted on top of the agar plate to detect light projected up through the agar from the stage light. Note that these values represent the total power of all wavelengths from UV to infrared. Values for specific colors would be much less (e.g. see Figure 5 for sunlight blue, blue-violet, and UV).

[1] This is the power used for the “dark” part of the light-dark locomotion assays.
[2] This is the power used for standard viewing of culture plates.
[3] This is the power received by culture plates on a bench from fluorescent room lighting.
[4]Based on data in Figure 4A.
[5] Measured at solar noon (12:45 p.m. on February 4, 2006) in Edmond, Oklahoma (elevation 1276 feet).
