# Supplementary material for: A Novel Molecular Solution for Ultraviolet Light Detection in Caenorhabditis elegans
Source: PLoS Biol. 2008 Aug 5;6(8):e198. doi: 10.1371/journal.pbio.0060198 (PMC2494560; doi:10.1371/journal.pbio.0060198)
Supplement: Supplementary file 10 [file pbio.0060198.st002.pdf]

Supplementary Table 2. *lite-1* mutations

Allele	Genomic sequence in N2 (wild type)	Genomic sequence in mutant	Effect on *lite-1* gene or protein
ce302	TTG **GT** AGT	TTT **AT** AGT	Splice donor site after end of 9th exon
ce303	CTG **GAT** TC	CTG **AAT** TC	D214N
ce304	CTC **TGG** GA	CTC **TGA** GA	W419 Stop
ce314	GCG **GT** ATT	GCG **AT** ATT	Splice donor site after end of 6th exon
ce318	CTT **CAG** GA	CTT **TAG** GA	Q106 Stop
ce319	TCT **CAA** AT	TCT **TAA** AT	Q376 Stop
ce320	TCC **AG** GGA	TCC **AA** GGA	Splice acceptor site before start of 9th exon
ce327	TCC **AG** TGT	TCC **AA** TGT	Splice acceptor site before start of 6th exon
ce329	TTA **CCA** GC	TTA **TCA** GC	P342S
ce338	CTT **TTG** GC	CTT **TTT G**GC	Insertion of T in 178th codon causing frameshift
ce340	TAT **CAA** AG	TAT **TAA** AG	Q276 Stop
ce341	TGC **CCG** CT	TGC **TCG** CT	P259S
ce342	AAT **CAA** TC	AAT **TAA** TC	Q62 Stop
ce343	TCC **CGT** GC	TCC **TGT** GC	R222C
ce345	TTG **AG** GTG	TTG **AA** GTG	S126N (mutation is in last base of exon; cDNA change is **AGC** to **AAC**)

ce312, ce316, and ce331, isolated from separate mutageneses, have the same mutation as *ce302*.
