# Supplementary material for: A Novel Molecular Solution for Ultraviolet Light Detection in Caenorhabditis elegans
Source: PLoS Biol. 2008 Aug 5;6(8):e198. doi: 10.1371/journal.pbio.0060198 (PMC2494560; doi:10.1371/journal.pbio.0060198)
Supplement: Supplementary file 11 [file pbio.0060198.sd001.doc]

## Text S1

## Worm culture and manipulation. Culture media (modified NGM, containing no added calcium or magnesium) consisted of the following (per liter): 2 g NaCl, 3.1 g Peptone, 3.0 g KH_2_PO_4_, 0.5 g K_2_HPO_4_, 20 g Sigma A-7002 agar. After autoclaving and cooling to 55˚ C the following was added (per liter): 1.6 ml 5 mg/ ml cholesterol in ethanol, 1.0 ml of 100 mg/ ml Streptomycin in ddH_2_O, 1.25 ml of 10 mg/ ml mycostatin suspension in ethanol. For “spread plates” we supplemented modified NGM with 1 g per liter of yeast extract and doubled the peptone. Worm food was strain OP-50 *E. coli* grown in “tryptone broth” (5 g NaCl; 10 g tryptone per liter) at 30˚ C overnight. Spread plates were spread with 100 ml of OP-50 culture 1 day after pouring and incubated 6 more days at room temperature. “Streak plates” were made by dipping a 6 mm rounded-tip glass rod into OP-50 culture and dragging once across the center of the plate for most of its diameter. The term “growing culture” used in the below methods means we plated five L2 larvae on spread plates and grew them 6d X 20˚ C to make adult F1 progeny that had never been starved.

**Strains.** Wild type strains were N2 (Bristol) [1] and CB4856 (Hawaiian) [2] as indicated. During outcrossing of *tax-2(p691)*, we discovered that the original strain contained a second site mutation in *lite-3*, which we designated *ce360*.

| **Strain name** | **Genotype** | **Reference** |
| --- | --- | --- |
| KG761 | *acy-1(pk1279); ceEx108 [myo-3::acy-1(+) cDNA]* | [3] |
| KG1272 | *ceIs37 [myo-3::lite-1 cDNA]* | This study |
| KG538 | *egl-30(ad805)* | [4] |
| KG1252 | *egl-30(ad805); lite-1(ce314)* | This study |
| KG1082 | *lite-1(ce302) 5x outcrossed* | This study |
| KG1076 | *lite-1(ce303) 1x outcrossed* | This study |
| KG1078 | *lite-1(ce304) 2x outcrossed* | This study |
| KG1092 | *lite-1(ce312) 0x outcrossed* | This study |
| KG1180 | *lite-1(ce314) 5x outcrossed* | This study |
| KG1108 | *lite-1(ce316) 2x outcrossed* | This study |
| KG1098 | *lite-1(ce318) 0x outcrossed* | This study |
| KG1099 | *lite-1(ce319) 0x outcrossed* | This study |
| KG1201 | *lite-1(ce320) 2x outcrossed* | This study |
| KG1130 | *lite-1(ce329) 2x outcrossed* | This study |
| KG1203 | *lite-1(ce331) 2x outcrossed* | This study |
| KG1133 | *lite-1(ce338) 2x outcrossed* | This study |
| KG1137 | *lite-1(ce340) 2x outcrossed* | This study |
| KG1138 | *lite-1(ce341) 2x outcrossed* | This study |
| KG1153 | *lite-1(ce342) 4x outcrossed* | This study |
| KG1202 | *lite-1(ce343) 2x outcrossed* | This study |
| KG1145 | *lite-1(ce345) 2x outcrossed* | This study |
| KG1154 | *lite-2(ce311) 5x outcrossed* | This study |
| KG1214 | *lite-3(ce360) 4x outcrossed* | This study |
| KG1259 | *lite-1(ce314); ceEx182 [rab-3::lite-1 cDNA]* | This study |
| KG1267 | *lite-1(ce314); ceEx186 [myo-3::lite-1 cDNA]* | This study |
| KG1268 | *lite-1(ce314); ceEx187 [unc-17::lite-1 cDNA]* | This study |
| KG1351 | *lite-1(ce314); ceEx190 [myo-3::GFP]* | This study |
| KG1271 | *lite-1(ce314); ceIs37 [myo-3::lite-1 cDNA]* | This study |
| KG1620 | *lite-1(ce314); ceIs51 [lite-1::GFP-lite-1 gene on fosmid]* | This study |
| KG1270 | *lite-2(ce311); ceEx189 [myo-3::lite-1 cDNA]* | This study |
| KG1269 | *lite-3(ce360); ceEx188 [myo-3::lite-1 cDNA]* | This study |
| KG552 | *unc-13(s69)* | [5] [6] |
| KG862 | *unc-31(e928)* | [7] |
| KG1178 | *unc-31(e928); lite-1(ce314)* | This study |

**Laser light source.** 405 nm, 5 mW laser with 0.9 mm diameter beam size (CrystaLaser; Reno, NV; #BCL-005-405) with CL2005 adjustable power supply. We mounted the laser head to a standard laboratory stand using a standard laboratory clamp. We positioned the laser to project onto the culture plate as near to vertical as possible in the center of the field of view while viewing animals through an Olympus SZX-12 stereomicroscope equipped with a 1.2X, 0.13 NA plan apochromatic objective.

**Mercury light source and light measurements.** We used the equipment shown and described in Figure S7 to project light of the desired wavelength and power through the stereomicroscope objective onto the culture plate surface. We used the standard Chroma filters shown in Figure 1B to restrict wavelengths to various ranges from UV to red. To measure the light power/ mm^2^ at the culture plate surface, we always placed the detector in a defined location/ orientation on the microscope stage due to inherent variation of different regions of the detector surface. We then zoomed to 108X to concentrate a light beam of 9.62 mm^2^ onto a subregion of the detector.

**Light-Dark Locomotion Assays.** After transferring a single healthy, mature adult from growing cultures to the center of each of ten room temperature locomotion plates, we waited >10 min before starting the assay. For the “dark” assay (the basal rate in the figures), we dimmed the ambient lighting and turned down the transmitted stage light as much as possible (to 70 nW/ mm^2^); however, we later found that ambient room and scope lighting has less than 1% of the minimum power needed to evoke the light response. We removed the plate lid and waited 30 sec before counting body bends for 6 minutes. A body bend is defined as each time the tip of the tail changes direction (i.e. when it reaches a maximum or a minimum in the waveform during forward or backward movement), with non-sinusoidal movements such as looping or coiling (which were rare in these light-dark assays) scored as described [8]. For the “light” assay, the light power was adjusted to the desired setting as described above and in Figure S7. The plate was placed on the stage (lid off) and, after 30 sec, the white stage light was increased to a level that allowed the viewer to clearly see the animal through the colored emission filter. After simultaneously switching the nosepiece slider to the desired filter and starting the timer for 6:30 min. body bends were counted during the final 6 min (i.e. there was a 30 sec equilibration in the light). We then re-checked and adjusted the light power before assaying the next animal, and repeated this until we had assayed 10 animals in this way. The data in Supplementary Fig. 9 represent 2 minute locomotion assays following a 30 second equilibration with an N of 20 animals.

We made the following modifications to the light-dark locomotion assay to quantify the light responses of the light hyper-responsive transgenic strains *lite-1(ce314); ceEx182* (*rab-3::lite-1* cDNA) and *lite-1(ce314); ceEx187* (*unc-17::lite-1* cDNA): 1) we used blue light from a GFP filter instead of the more potent blue-violet light from a CFP filter; 2) for *ceEx182*, the bulb was producing 2620 μW/ mm^2^ of blue power at 108X, and we zoomed the magnification out to 19.2X, thus producing an estimated blue light power of ~466 μW/ mm^2^. This is ~17% of optimal power of blue light for wild type. At the time of the *ceEx187* (*unc-17::lite-1* cDNA) assay, the bulb was producing 2017 μW/ mm^2^ of blue power at 108X, and we zoomed the magnification out to 15X, thus producing an estimated blue light power of ~280 μW/ mm^2^. This is ~10% of optimal power of blue light for wild type; 3) Figure 8A shows the locomotion rate of *ceEx182* only for the first minute of illumination after the 30 second equilibration to the light (which we determined to be the peak interval in a 6 minute assay). Figure 8B shows the locomotion rate of *ceEx187* for the one minute interval from 2 – 3 min after the start of illumination (which we determined to be the peak interval).

For regional illumination “head light” assays in Fig. 9B, we set up the 405 nm laser to project into the field of view of the stereomicroscope and adjusted the power to 5.0 mW. Under regular transmitted light, 9 mature adult *unc-31(e928)* or 6 *egl-30(ad805)* were transferred from a growing culture to each of 10 locomotion plates, laying the animals down in different parts of the plate. After letting the animals equilibrate for 10 minutes with the plate lids off, we selected one of the animals on the first plate and oriented the animal as described for the above reversal assay. Keeping the laser spot a half body length from the head of the animal, we started a timer and counted body bends for 1 min. This represents the “dark” or low white light control assay. We then moved the plate such that the laser illuminated the animal’s head region (down to the posterior bulb of the pharynx) and simultaneously started a timer for 30 sec (*unc-31(e928)*) or 1 min (*egl-30(ad805)*) and counted body bends during this time. *unc-31* was assayed for a shorter time because, after 30 seconds, the animals tended to double back on itself, thus making it harder to maintain head-only illumination. If this happened during the 30 sec, we cancelled the assay for the animal, picked it off, and we chose another. Upon completion of the assay, we picked the animal off of the plate and repeated the assay with another animal on the plate, cycling through the 10 plates until we had assayed 60 (*unc-31(e928)*) or 30 (*egl-30(ad805)*) animals. We performed the regional illumination “tail light” assays in Figure 9B similarly, except we illuminated the tail region from ~preanal ganglion to the tip of the tail, and we assayed 30 animals of each genotype (N2, *e928*, and *ad805*) for 1 min each after a 20 second equilibration to the light (we did not count body bends during the first 20 sec of illumination, in an effort to mimic the assay conditions of the standard Light-Dark locomotion assay using whole body illumination). To quantify the initial direction of movement in response to head or tail illumination (i.e. Figure 9A and the text), we produced growing cultures on spread plates (several hundred animals per plate). With the plate lid off, we scanned the plate for animals not moving and then moved the plate such that the desired region entered the laser spot on the plate surface and noted the response during a 2 second interval. For initial direction of movement in response to whole animal illumination, we used the stereomicroscope and CFP filter set with the light power on 1500 μW/ mm^2^, moving the filter into the light path after finding an immobile animal.

**Temperature experiments.** To measure temperature changes induced by direct illumination of a metal probe on the culture plate surface, we first adjusted the light source to the proper wavelength and power/ mm^2^ as described above and in Figure S7, and then moved the light path back to white stage light and placed an unseeded culture plate on the stage with its lid off. We secured the metal probe of a Checktemp1 digital thermometer (HANNA Instruments) such that the beveled edge of the probe rested flat on the surface of the plate in the center. We zoomed to 108X and focused on the probe tip and make sure it is in the center of the field of view and that the light beam/ field of view is restricted to the beveled edge of the tip. After noting the initial temperature reading, we simultaneously started a timer for 2 min and switched the light path to the desired filter. After 2 min, we noted the temperature reading (we had previously found that the temperature does not change significantly after 2 min). We then switched the light path back to white stage light and allowed the probe to re-equilibrate to the t = 0 point +/- 0.1˚ C before repeating the experiment 2 more times for each color.

To measure temperature changes induced by illumination of the probe in M9 buffer or a worm pellet, we transferred five L2 stage larvae to each of 8 streak plates and grew them 7 d at room temperature to produce young larvae on freshly starved plates. We then plated 5000 larvae on each of 19 spread plates and grew them 2 d at room temperature to produce adults. We rinsed these off of the plates with M9 buffer into 15 ml conicals, pelleted 1 min X 1500 rpm, washed once with 12 mls of M9, combined the pellets into 1 tube and respun to remove as much buffer as possible. We then used a blunt-cut P200 tip to transfer 50 μl of worm pellet to each of 9 X 200 μl thin-wall PCR tubes. For controls, we transferred 50 μl M9 into each of 9 X 200 thin-wall PCR tubes. We adjusted the light source proper wavelength and power/ mm^2^ as described above and in Figure S7. We arranged the temperature probe so the tip rested at ~10 degree angle relative to the stage surface. We then inserted the probe all of the way into the first tube until it touched the tube bottom and then laid the tube with the inserted probe flat on the stage. We zoomed to 108X and focused on tube tip such that the rounded part of the tube was exactly at the left part of the field of view. We then pulled the metal probe tip back slightly from the bottom of the tube, so it was not exposed by displacing the worm suspension at the tip of the tube. After stabilizing the probe at ambient room temperature (20.4˚ C for this experiment), we simultaneously started the timer and switched to the desired color. After 2 min, we recorded the temperature, re-equilibrated the probe to 20.4 +/- 0.1˚ C, and repeated additional trials and conditions as described.

To test the effect of temperature on the locomotion rate of the *unc-31* null, we simultaneously equilibrated two locomotion assay plates (1 experimental plate and 1 duplicate dummy control) for 2 h in a Bellco Autoblot Hybridization oven set at the desired temperature and monitored with an internal thermometer. We placed each plate lid side down in the lid of a Sarstedt styrofoam box for microtubes (20 – place size) during the equilibration. At the end of the 2 hours, we quickly transferred the prewarmed dummy culture plate to the scope. We placed the dummy plate on an inverted Petri plate lid on the scope stage to slow heat transfer. We then Immediately inserted the tip of the Checktemp 1 metal probe all of the way into the agar, moving it to a new location at 10, 20, and 30 seconds to allow the probe tip to warm to the plate temperature. We then kept the probe in the final position for 1 min and recorded the temperature at the beginning and end of the minute. We called the average of these two temperatures the “plate surface temperature”. For the actual assay, we pre-transferred 10 mature adult *unc-31* null mutants from a growing culture to an intermediate “transfer plate” so that they were close together and ready to transfer to the heated experimental plate. We placed this transfer plate on top of an inverted Petri lid and focused the scope on the 10 animals so they would be in the same plane of focus as the heated experimental plate. We then transferred the heated experimental plate to a separate inverted Petri lid on the microscope stage and started a timer. During the first 30 sec, we transferred the 10 animals from the transfer plate to the preheated plate and used the pick to “erase” any marks from the plating that could look like tracks. The animals lay undisturbed from 30 sec – 1 min 30 sec after plating. At 1 min 30 sec we transferred the plate to a dry ice block (smooth side down) for 1 min (with the lid off, because the CO_2_ as well as the cold stops any movement immediately). We then simply observed the tracks of each animal and noted the number of body bends (sinusoidal peaks or valleys) that each animal made during its 1 min incubation on the heated experimental plate.

**Time course assays.** From growing cultures, we plated 10 healthy mature adults on separate locomotion plates and allowed the animals to settle at least 15 min before the assay. We adjusted the light power to the desired setting as described above and in Figure S7. Then with the light path set to a level of white light that allows the viewer to clearly see the animal when the colored light comes on and the plate lid off, we located the animal on the first plate, zoomed to 108X magnification, and waited until the animal stopped moving (*unc-31* null) or slow down to <= 1 BB every 5 seconds (N2). We used two people and multiple finger counters for this assay. Person 1 moved the slider to the appropriate light filter and counted body bends on a finger counter. Person 2 started a count up timer when the light pathway was switched to the colored light filter, switched finger counters at each time point, recorded data for each interval, and reset finger counters for re-use. These methods apply to both the “Time course of response and decay of response” experiments and the “Extended time course” (30 min) assays. For the 1h light damage time course assays, we plated a single healthy mature adult on a each of two locomotion plates (1 for experimental and1 for the unilluminated control) and allowed animals to settle 10 min before the assay. We adjusted the light power to the desired setting as described above and in Figure S7. Then with the light path set to low power white light and the plate lid off, we located the animal on the first plate, zoomed to 108X magnification, and waited 30 sec. We then counted body bends for 30 seconds and recorded this basal rate data. We then turned up the white light power to a level that will allows one to clearly see the animal in a colored light background. We simultaneously switched to colored light and started a 30 second count down timer and a count up timer. We counted body bends during the initial 30 seconds of exposure and recorded this data. We then continued to keep the animal in the light. From 4:30 – 5:30 min, we counted body bends and then at 5 min intervals thereafter until 1 hour. After 1 hour, we switched off the colored light, replaced the plate lids, and kept the count up timer going. We tested all animals that appeared dead at the end of 1 h of light exposure (i.e. all animals exposed to blue-violet light) for signs of life at 2 h, 4 h, 24 h, and 48 h by observing pharyngeal pumping, signs of tracks, and response to harsh touch. None of the blue-violet exposed animals showed any sign of life by these criteria.

##	DNA constructs. To make KG#245 (*unc-17β::lite-1* cDNA), we used StrataScript reverse transcriptase (Stratagene) and a primer engineered with a restriction site to transcribe a full-length *lite-1* cDNA template from purified wild type *C. elegans* mRNA. We then used Accuprime Pfx (Invitrogen) and primers engineered with restriction sites to amplify and clone the cDNA into the multi-cloning site of Nhe I/ Age I cut KG#65 (an unc-17β:: *C. elegans* expression vector described in [7]). To make KG#250 (*rab-3::lite-1* cDNA), we used Nhe I/ Age I to remove the *lite-1* cDNA from KG#245 (*unc-17β::lite-1* cDNA) and cloned this fragment into like-digested KG#59 (*rab-3*::, a pan-neuronal *C. elegans* expression vector described in [8]). RM#349p (*unc-17*::GFP) is a gift from Jim Rand. pPD96.52 (*myo-3::* expression vector) and pPD118.20 are gifts from Andrew Fire. To make KG#94 (*unc-17::* expression vector), we used Apa I/ Msc I to cut out the ~1900 bp multi-cloning site + GFP + *unc-54* 3’ control region from RM#349p (*unc-17*::GFP), leaving the 5900 bp vector fragment containing the 3.2 Kb *unc-17* promoter. To this fragment we ligated the 1079 bp Msc I/ Apa I fragment (containing the multi-cloning site and *unc-54* 3’ control region) cut from pPD96.52 (*myo-3::* expression vector). To make KG#270 (*unc-17::lite-1* cDNA), we used Nhe I/ Age I to remove the *lite-1* cDNA from KG#250 and cloned it into like-digested KG#94 (*unc-17::* expression vector). To make KG#269, we used Nhe I/ Age I to cut out the 1400 bp lite-1 cDNA from KG#250 and cloned it into like-digested pPD96.52 (*myo-3::* expression vector).

**Transgenes.** We made *lite-1(ce314); ceEx190* by injecting *lite-1(ce314)* with pPD118.20 (*myo-3*::GFP) at 15 ng/ μl. We made *lite-1(ce314); ceEx186*, *lite-2(ce311); ceEx189*, and *lite-3(ce360); ceEx188* by injecting each mutant with KG#269 (*myo-3::lite-1* cDNA) at 15 ng/ μl along with the co-transformation marker pPD118.20 (myo-3::GFP at 15 ng/ μl. We made *lite-1(ce314); ceEx182* (*rab-3::lite-1* cDNA) by injecting *lite-1(ce314)* with KG#250 at 12.5 ng/ μl along with the co-transformation markers KG#67 (*ttx-3::*GFP) and KG#68 (*rab-3::*GFP) at 15 ng/ μl each. We made *lite-1(ce314); ceEx187* (*unc-17::lite-1* cDNA) by injecting *lite-1(ce314)* with KG#270 at 25 ng/ μl along with the co-transformation marker RM#349p (*unc-17::*GFP) at 20 ng/ μl. We made *lite-1(ce314); ceEx216* [GFP-*lite-1* on fosmid 29B12] by injecting *lite-1(ce314)* with KG#319 (see Recombineering) at 1 ng/ μl along with the co-transformation markers KG#255 (*ttx-3::*RFP) and KG#247 (*rab-3::*RFP) at 12 ng/ μl each. *ceIs37* [myo-3::lite-1 cDNA] and *ceIs51* [GFP-lite-1 on fosmid 29B12] are integrated versions of the *ceEx186* and *ceEx216* arrays.

## Text S1 References

1. Brenner S (1974) The genetics of *C. elegans*. Genetics 77: 71-94.

2. Wicks SR, Yeh RT, Gish WR, Waterston RH, Plasterk RH (2001) Rapid gene mapping in Caenorhabditis elegans using a high density polymorphism map. Nat Genet 28: 160-164.

3. Reynolds NK, Schade MA, Miller KG (2005) Convergent, RIC-8 Dependent Gα Signaling Pathways in the *C. elegans* Synaptic Signaling Network. Genetics 169: 650-670.

4. Brundage L, Avery L, Katz A, Kim U, Mendel JE, et al. (1996) Mutations in a *C. elegans* G_q_α gene disrupt movement, egg laying, and viability. Neuron 16: 999-1009.

5. Kohn RE, Duerr JS, McManus J, Duke A, Rakow TL, et al. (2000) Expression of multiple UNC-13 proteins in the *Caenorhabditis elegans* nervous system. Molecular Biology of the Cell 11: 3441-3452.

6. Richmond JE, Davis WS, Jorgensen EM (1999) UNC-13 is required for synaptic vesicle fusion in *C. elegans*. Nature Neuroscience 2 (11): 959-964.

7. Charlie NK, Schade MA, Thomure AM, Miller KG (2006) Presynaptic UNC-31 (CAPS) is Required to Activate the Gα_s_ Pathway of the Synaptic Signaling Network. Genetics 172: 943-961.

8. Schade MA, Reynolds NK, Dollins CM, Miller KG (2005) Mutations that Rescue the Paralysis of *C. elegans* *ric-8* (Synembryn) Mutants Activate the Gα_s_ Pathway and Define a Third Major Branch of the Synaptic Signaling Network. Genetics 169: 631-649.
